# GRP78 Nanobody‐Directed Immunotoxin Activates Innate Immunity Through STING Pathway to Synergize Tumor Immunotherapy

**DOI:** 10.1002/advs.202408086

**Published:** 2025-03-26

**Authors:** Huifang Wang, Runhua Zhou, Chengchao Xu, Lingyun Dai, Rui Hou, Liuhai Zheng, Chunjin Fu, Guangwei Shi, Jingwei Wang, Yang Li, Jinpeng Cen, Xiaolong Xu, Le Yu, Yilei Li, Jigang Wang, Qingfeng Du, Zhijie Li

**Affiliations:** ^1^ Department of Critical Care Medicine Guangdong Provincial Clinical Research Center for Geriatrics Shenzhen Clinical Research Centre for Geriatrics Department of Nuclear Medicine Shenzhen People's Hospital (The First Affiliated Hospital, Southern University of Science and Technology; The Second Clinical Medical CollegeJinan University) Shenzhen Guangdong 518020 China; ^2^ Post‐doctoral Scientific Research Station of Basic Medicine Jinan University Guangzhou 510632 China; ^3^ Clinical Pharmacy Center Nanfang Hospital Southern Medical University Guangzhou Guangdong 510515 China; ^4^ Harry Perkins Institute of Medical Research QEII Medical Centre and Centre for Medical Research The University of Western Australia Nedlands WA 6009 Australia; ^5^ Department of Neurosurgery & Medical Research Center Shunde Hospital Southern Medical University (The First People's Hospital of Shunde Foshan) Guangzhou 510515 China; ^6^ Department of Urology Nanfang Hospital Southern Medical University Guangzhou Guangdong 510515 China; ^7^ School of Traditional Chinese Medicine and School of Pharmaceutical Sciences Guangdong Provincial Key Laboratory of New Drug Screening School of Pharmaceutical Sciences Southern Medical University Guangzhou Guangdong 510515 China; ^8^ State Key Laboratory for Quality Ensurance and Sustainable Use of Dao‐di Herbs Artemisinin Research Center Institute of Chinese Materia Medica China Academy of Chinese Medical Sciences Beijing 100700 China; ^9^ State Key Laboratory of Antiviral Drugs School of Pharmacy Henan University Kaifeng 475004 China

**Keywords:** cell surface glucose‐regulated protein 78 (GRP78), immunotherapy, immunotoxin, STING pathway, targeted therapy

## Abstract

The lack of targetable antigens poses a significant challenge in developing effective cancer‐targeted therapies. Cell surface translocation of endoplasmic reticulum (ER) chaperones, such as glucose‐regulated protein 78 (GRP78), during malignancy, drug resistance, and ER stress induced by therapies, offers a promising pan‐cancer target. To target GRP78, nanobody C5, identified from a phage library and exhibiting high affinity for human and mouse GRP78, is utilized to develop the *Pseudomonas* exotoxin (PE) immunotoxin C5‐PE38. C5‐PE38 induced ER stress, apoptosis and immunogenic cell death in targeted cells and showed antitumor efficacy against colorectal cancer and melanoma models without obvious toxicity. Mechanistically, transcriptome profiling showed that C5‐PE38 reshaped the tumor immune microenvironment with enhanced innate and adaptive immune response and response to interferon beta. Moreover, C5‐PE38‐induced cell death could trans‐activate STING pathway in dendritic cells and macrophages, promoting CD8^+^ T cell infiltration. It also sensitizes both primary and metastatic melanomas to anti‐PD1 therapy, partly through STING activation. Overall, this study unveils a feasible GRP78 nanobody‐directed therapy strategy for single or combinatorial cancer intervention. This work finds that C5‐PE38‐induced cell death stimulates STING‐dependent cytosolic DNA release to promote antitumor immunity, a mechanism not previously reported for PE38, providing valuable insights for its clinical use.

## Graphical abstract

A GRP78 nanobody‐directed immunotoxin suppresses cancer progression and metastasis by enhancing antitumor immunity via STING pathway activation, offering a pan‐cancer‐targeted approach and immunotherapy combination strategy.

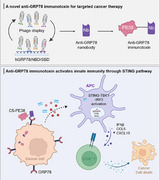

## 1 Introduction

Cancer remains a highly lethal disease worldwide. Conventional cancer treatments, involving physical and chemical interventions, are often associated with recurrence, drug resistance, and metastasis. Moreover, these therapies cannot accurately differentiate between malignant cells and healthy ones, giving rise to numerous undesired side effects. Significant strides have been made in cancer therapy over the past few decades, particularly in the realms of immunotherapy and antibody‐based targeted therapy.^[^ ^1^ ^]^ Antibody‐based targeted therapies, such as monoclonal antibodies, antibody‐drug conjugates (ADCs), and bispecific antibodies, aim to specifically suppress cancer cells while maintaining the normal tissues intact. However, the development of antibody‐based targeted therapy is still hindered by limited efficacy resulting from drug resistance (antigen loss), safety concerns, and the uneven presence of a small range of tumor antigens that can be targeted.^[^ ^2^ ^]^ Human epidermal growth factor receptor 2 (HER2), nectin‐4, trophoblast surface antigen 2 (TROP2), folate receptor alpha (FRα), and tissue factor (TF) have been validated as antibody targets for solid‐tumor therapy.^[^ ^2^, ^3^ ^]^ Emerging various tumor targets are also demonstrated to be heterogeneously expressed in tumor tissues. Meanwhile, many of these targets are limited to a few cancer types, emphasizing the urgent need to discover universal tumor‐specific antigens that are expressed across a wide range of tumors.

Glucose‐regulated protein 78 (GRP78), also known as binding immunoglobulin protein (BiP) and encoded by the HSPA5 gene, is a master regulator of the unfolded protein response (UPR) and is primarily localized within the endoplasmic reticulum (ER).^[^ ^4^ ^]^ However, in response to elevated ER stress, GRP78 is upregulated and translocated to various cellular compartments such as the nucleus, mitochondria, and even the cell membrane surface.^[^ ^5^ ^]^ The cell surface localization of GRP78 was initially reported in malignant T lymphocytes in 1997.^[^ ^6^ ^]^ Since then, cell surface GRP78 (csGRP78) has been found to be expressed in a broad range of malignancies, including both solid tumors such as glioblastoma,^[^ ^7^ ^]^ pancreatic cancer,^[^ ^8^ ^]^ breast cancer,^[^ ^9^ ^]^ gastric cancer,^[^ ^10^ ^]^ prostate cancer,^[^ ^11^ ^]^ lung cancer,^[^ ^12^ ^]^ and hematological tumors like acute myeloid leukemia.^[^ ^13^ ^]^ In addition to ER stress, various stress conditions such as nutritional stress, hypoxia, radiation therapy, chemotherapy, and drug resistance can induce the expression of csGRP78 in cancer cells.^[^ ^14^ ^]^ csGRP78 is also overexpressed in cancer stem cells and tumor‐associated endothelium.^[^ ^7^, ^15^ ^]^ These findings indicate csGRP78 as a potential pan‐cancer target applicable to the heterogeneous tumor microenvironment (TME). Importantly, csGRP78 is never or rarely manifested on the exterior of normal cells.^[^ ^5a^ ^]^ And as a common essential gene according to DepMap Portal, GRP78 is less prone to antigen loss, which may reduce resistance development for drugs targeting csGRP78. Moreover, the varied mechanisms driving the cell surface translocation of GRP78 in tumor cells offer great opportunities to develop therapeutic strategies against csGRP78.^[^ ^16^ ^]^ All these characteristics render GRP78 an exceptionally appealing target antigen for tumor therapies.

Targeting ligands, including antibodies and peptides, have been utilized to specifically target csGRP78 and to deliver cargoes of interest directly to tumor tissues. For instance, synthetic chimeric peptides containing a GRP78‐binding motif fused to proapoptotic peptides inhibit tumor growth without affecting normal tissues.^[^ ^17^ ^]^ The micelles, modified with a csGRP78‐targeting peptide, along with temozolomide and paclitaxel, demonstrated cytotoxicity against glioma tumor cells and stem cells.^[^ ^18^ ^]^ Furthermore, csGRP78‐targeted CAR‐T cells eradicated acute myeloid leukemia, and multiple solid tumors, and extended the life of mice.^[^ ^13^, ^19^ ^]^ Another major advance is that certain nake antibodies against csGRP78 exhibit therapeutic efficacy alone through diverse mechanisms.^[^ ^14^ ^]^

Immunotoxins represent a class of immunoconjugates, which are not strictly ADCs and consist of a plant or bacterial toxin rather than a chemotherapeutic agent. Moxetumomab pasudotox, a *Pseudomonas* exotoxin (PE)‐based immunotoxin targeting CD22, achieved highly durable complete responses in hairy cell leukemia and received approval from the US FDA for this indication in 2018.^[^ ^20^ ^]^ PE‐based immunotoxins employ a unique mechanism of cell killing, which is distinct from that of currently approved chemotherapies. The toxin catalyzes irreversible adenosine diphosphate (ADP)‐ribosylation of the diphthamide residue of eukaryotic translation elongation factor 2 (EF2), resulting in protein synthesis arrest and ultimately cell death.^[^ ^21^ ^]^ Due to their high specificity and effectiveness, PE‐based immunotoxins are increasingly utilized in clinical development and research studies. Many popular antigens including mesothelin, Lewis Y antigen, Interleukin‐4, CD25, Epidermal Growth Factor Receptor, HER2, Vascular Endothelial Growth Factor Receptor 2, glypican‐3, B‐Cell Maturation Antigen, and transferrin receptor were used as action targets for a variety of cancer diseases.^[^ ^22^ ^]^ To our knowledge, there are no reported immunotoxins targeting GRP78 or other translocated ER chaperones.

Although immunotoxins have well‐established anti‐cancer effects by inhibiting protein synthesis, emerging clinical evidence suggests that they can also provoke anti‐tumor immunity.^[^ ^23^ ^]^ For instance, some patients treated with Moxetumomab pasudotox experienced delayed complete regression 6 months after discontinuation of treatment,^[^ ^20^ ^]^ indicating potent immunomodulatory effects beyond direct tumor cell killing. Similar delayed responses have been noted in patients treated with other PE immunotoxins targeting antigens like mesothelin (SS1P), EpCAM (VB4‐845), and HER2 (scFv (FRP5)‐ETA).^[^ ^24^ ^]^ These observations are reminiscent of patterns seen with immune checkpoint inhibitors,^[^ ^25^ ^]^ suggesting a potential role for immunotoxins in stimulating anti‐tumor immunity. Moreover, recent research has shown that LMB‐100, an anti‐mesothelioma PE24, triggered a systemic inflammatory response in some patients, and improved progression‐free survival when combined with pembrolizumab, an antibody against programmed cell death 1 (PD‐1).^[^ ^26^ ^]^ These findings collectively highlight a broader immunomodulatory role for PE immunotoxins in clinical settings. Thus, numerous studies are investigating the immune‐related mechanisms of PE immunotoxins. However, it remains incompletely understood at present.

In this study, we fused a novel GRP78‐binding nanobody (Nb) C5, identified from an alpaca Nb phage display library, with a truncated PE toxin (PE38) to develop a recombinant immunotoxin against scGRP78. The resulting C5‐PE38 immunotoxin exhibited significant antitumor activity both in vitro and in vivo in tumors expressing csGRP78. Additionally, we investigated the immunomodulatory effects of C5‐PE38 in an immunocompetent melanoma model. We found that C5‐PE38 reshapes the tumor microenvironment and induces innate and adaptive immune responses via the Stimulator of Interferon Genes (STING) pathway. Furthermore, we demonstrated that combining C5‐PE38 with an anti‐PD1 antibody led to an augmented antitumor immune response, effectively controlling primary tumor growth and metastasis of melanoma. This study underscores the potential of csGRP78 as a promising pan‐cancer target for immunotoxin therapy. It also highlights the ability of PE immunotoxins to indirectly activate the STING pathway, thereby aiding in the establishment of anti‐tumor immunity.

## 2 Results

### 2.1 GRP78 is Expressed on the Cell Surface of Multiple Solid Tumors

**Figure 1:**
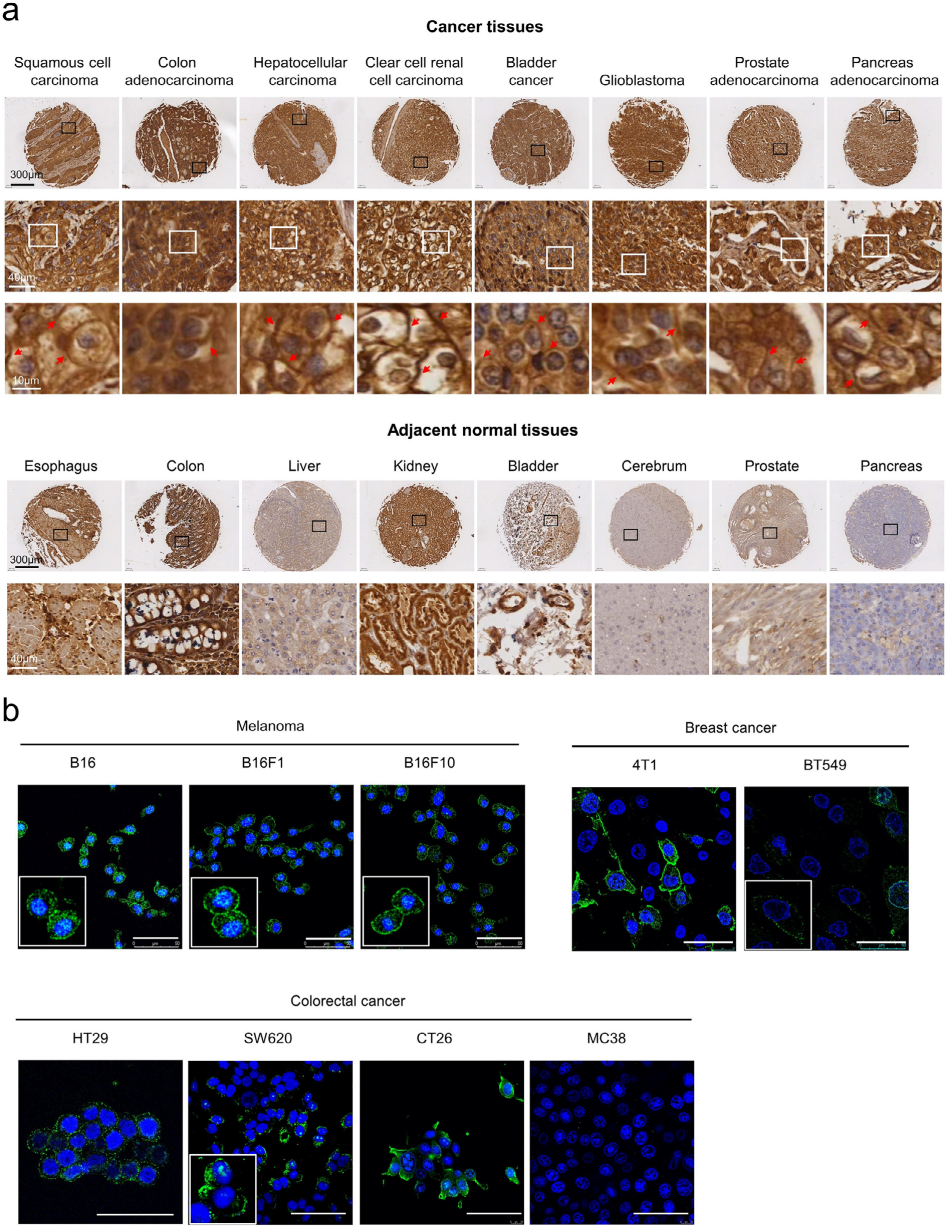
GRP78 is overexpressed on the cell surface across various solid malignancies. a) Representative immunohistochemistry (IHC) staining for GRP78 illustrating elevated surface GRP78 expression in pan‐cancer tissues compared to low surface GRP78 expression in adjacent normal tissues. Scale bars, 300 µm, 40 µm, 10 µm (low to high magnification). The red arrows indicated the csGRP78. b) Representative immunofluorescence staining of csGRP78 in melanoma, breast cancer, and colorectal cancer cell lines. Scale bars, 50 µm.

It has been previously reported that GRP78 (HSPA5) is commonly expressed in many types of cancer. Consistent with these reports, our analysis of HSPA5 gene expression using the Cancer Genome Atlas (TCGA) and the Genotype‐Tissue Expression (GTEx) database (Gepia2)^[^ ^27^ ^]^ shows that HSPA5 was highly expressed across multiple cancers (Figure S1, Supporting Information). Furthermore, analysis of the Clinical Proteomic Tumor Analysis Consortium (CPTAC) dataset^[^ ^28^ ^]^ revealed significantly higher HSPA5 protein expression in tumor tissues compared to normal tissues in several cancer types (Figure S2, Supporting Information). In addition, higher expression levels were associated with advanced pathological stages in certain cancers (Figure S3, Supporting Information). Kaplan‐Meier survival curves showed that patients with high HSPA5 expression had significantly diminished overall survival (OS) compared to those with low expression in adenoid cystic carcinoma (ACC), bladder cancer (BLCA), cervical squamous cell carcinoma and endocervical adenocarcinoma (CESC), glioblastoma (GBM), liver hepatocellular carcinoma (LIHC), and uveal melanoma (UVM) (Figure S4, Supporting Information). These findings suggest that GRP78/HSPA5 may serve as a potential biomarker and therapeutic target for multiple cancer types. ER stress triggers the UPR, resulting in elevated expression of GRP78 and its translocation to the cell surface.^[^ ^5b^ ^]^ Numerous studies support the role of csGRP78 as a hallmark of cancers, including ovarian, prostate, brain, breast cancer, myeloma, melanoma, and lymphoma, as well as other stressed cells.^[^ ^5^, ^14^ ^]^ To further confirm GRP78 as a potential target for cancer therapy, the expression of GRP78 was examined via immunohistochemical staining on a tissue microarray encompassing eight different cancer types and their adjacent tissues. The result indicated that GRP78 was predominantly abundant in the cytosol of most cancer tissues, with clear positive staining on the cell membrane (**Figure** **1**a). Importantly, there were nearly no detectable csGRP78‐positive signals in the adjacent normal tissues, despite the presence of low or moderate expression of cytosolic GRP78 (Figure 1a). Additionally, immunofluorescent staining of GRP78 without permeability was used to assess cell‐surface expression of GRP78 in several melanoma (B16, B16F1, B16F10), colorectal (HT29, SW620, CT26, MC38), and breast cancer cell lines (4T1, BT549). The results revealed a hierarchy of csGRP78 expression in the tested cells: high‐csGRP78‐expressing cells (B16, B16F1, B16F10, HT29, CT26), middle‐expressing cells (4T1, SW620), and low‐expressing cells (MC38, BT549) (Figure 1b). As an example, co‐localization staining in HT29 cells clearly demonstrated the distinct presence of GRP78 on the cell surface (Figure S5, Supporting Information). Collectively, these data suggest the translational potential of csGRP78 as a promising biomarker and therapeutic target for multiple cancer types.

### 2.2 Screening Nanobodies Binding to GRP78

**Figure 2:**
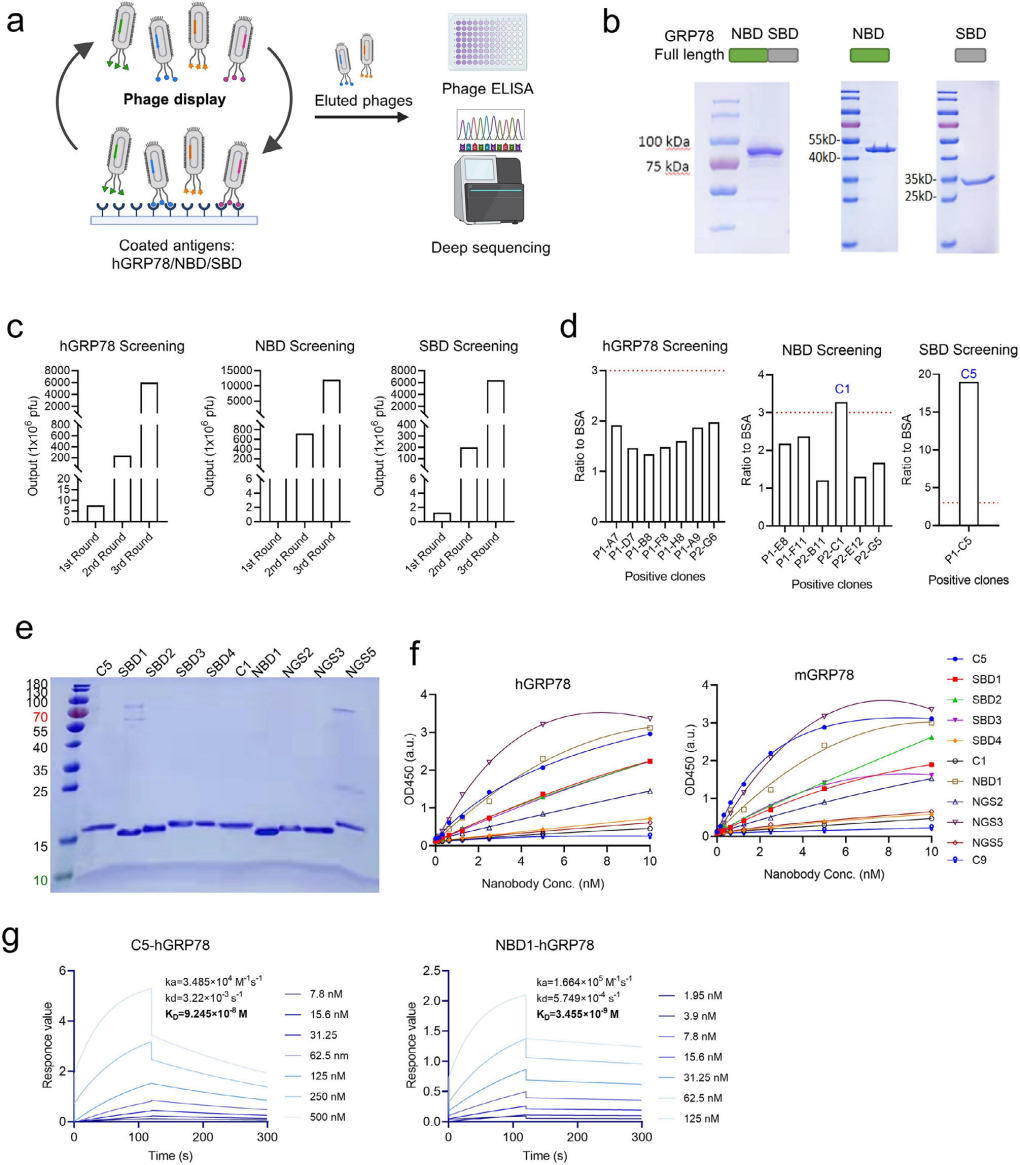
Selection of GRP78‐specific nanobodies (Nbs) from a naïve phage Nb library. a) Schematic of screening GRP78‐binding Nbs using phage display biopanning, phage ELISA, and deep sequencing analysis. b) SDS‐PAGE analysis of purified full‐length GRP78 and its major domains: NBD and SBD. c) Phage recovery during Nb screening, showing enrichment with nearly 800‐, 1000‐, and 5000‐fold increases for full‐length hGRP78, NBD domain, and SBD domain screening, respectively, after 3 rounds of screening. d) Phage ELISA against three GRP78 fragment antigens. Positive clones were identified based on an OD450 ratio of antigen to BSA ≥3. Based on this standard, one positive clone was identified for NBD domain and SBD domain, respectively, and no positive binder was found for full‐length hGRP78. e) Ten Nbs identified from phage ELISA and deep sequencing were purified and examined by SDS‐PAGE. f) ELISA analysis evaluated the binding ability of the 10 Nbs and an irrelevant control Nb (C9) to human GRP78 (hGRP78) and mouse GRP78 (mGRP78). g) Affinity determination of Nb C5 and Nb NBD1 binding to immobilized hGRP78 using surface plasmon resonance (SPR) assay. The equilibrium dissociation constant (K_D_), association rate constant (k_a_), and dissociation rate constant (k_d_) are indicated in each graph.

GRP78 is a 78 kDa protein comprising two major domains: the nucleotide‐binding domain (NBD), responsible for ATP capture and hydrolysis, and the substrate‐binding domain (SBD), facilitating interactions with other proteins.^[^ ^5a^ ^]^ To identify high‐affinity Nbs against GRP78, both full‐length and fragments were utilized for screening using a naïve alpaca phage Nb display library (**Figure** **2**a). The human GRP78 (hGRP78) and its two domains were purified and yielded highly pure proteins (Figure 2b). Each antigen underwent three consecutive rounds of biopanning. Remarkably, the phage clones specific to hGRP78, NBD, and SBD were effectively enriched by ≈800‐, 1000‐, and 5000‐fold, respectively (Figure 2c), indicating successful amplification of potential binders. 192 phage clones from the third round of sub‐library were randomly selected and screened for specific binding to the original antigens using phage enzyme‐linked immunosorbent assay (ELISA). Through phage ELISA and sequencing alignment, only one potentially positive binder (highlighted in blue, C1 for NBD and C5 for SBD) was identified from both the NBD and SBD screenings, while no binder with high activity was discovered from the hGRP78 screening (Figure 2d). However, phage ELISA experiments examined only a limited number of clones, insufficient to reflect the overall frequency distribution of clones in the screened library. Therefore, we performed deep sequencing of the Nb sub‐library obtained from the third round of screening of the three antigens. After deep sequencing, over twenty million independent Nb sequences were obtained. Nb sequences were ranked based on frequencies, and sequences with a frequency ≥ 0.9% were selected for further assessment. The selected sequences were named and highlighted in blue in Figure S6, Supporting Information, with five sequences (NGS1, NGS2, NGS3, NGS4, NGS5) from hGRP78 deep sequencing, four (SBD1, SBD2, SBD3, SBD4) from SBD deep sequencing, and one (NBD1) from NBD deep sequencing. Among them, the NGS1 sequence is identical to SBD1. In total, we obtained 11 potent binders from the three antigen screenings: 2 from phage ELISA identification and 9 from deep sequencing.

To evaluate the binding capability of the Nbs against GRP78, we expressed the His and HA tag‐labeled Nbs in E. coli BL21 (DE3) cells and purified them using the Ni‐NTA column. SDS‐PAGE analysis revealed that ten Nbs, except NGS4, were purified with the expected apparent molecular weight (≈15 kDa) (Figure 2e). The complementarity determining region (CDR) sequences of these Nbs are presented in Table S1, Supporting Information. Subsequently, we confirmed the binding ability of the Nbs against GRP78 using ELISA, with an irrelevant Nb (C9) as a negative control. Given the high sequence conservation (99%^[^ ^29^ ^]^) between human and murine GRP78 (mGRP78), we also tested the binding ability of these Nbs to mGRP78. Results showed that NGS3 from hGRP78 deep sequencing, C5 from SBD phage ELISA, and NBD1 from NBD deep sequencing exhibited the highest binding activity to both hGRP78 and mGRP78, while the control Nb C9 did not demonstrate any binding activity (Figure 2f). Moreover, none of the Nbs exhibited binding activity toward the BSA protein (Figure S7, Supporting Information). Nb NGS3 was excluded from the following study due to low yield. To assess the binding affinity, a surface plasmon resonance (SPR) experiment was conducted. Results showed that Nb C5 and Nb NBD1 had an excellent affinity to hGRP78 (K_D_ = 92 and 3.4 nM, respectively) (Figure 2g). Overall, we screened and obtained a panel of Nbs capable of binding to both human and murine GRP78, potentially expanding the therapeutic applications based on csGRP78.

### 2.3 Nb C5 Targets Tumors with csGRP78 Expression Both In Vitro and In Vivo

**Figure 3:**
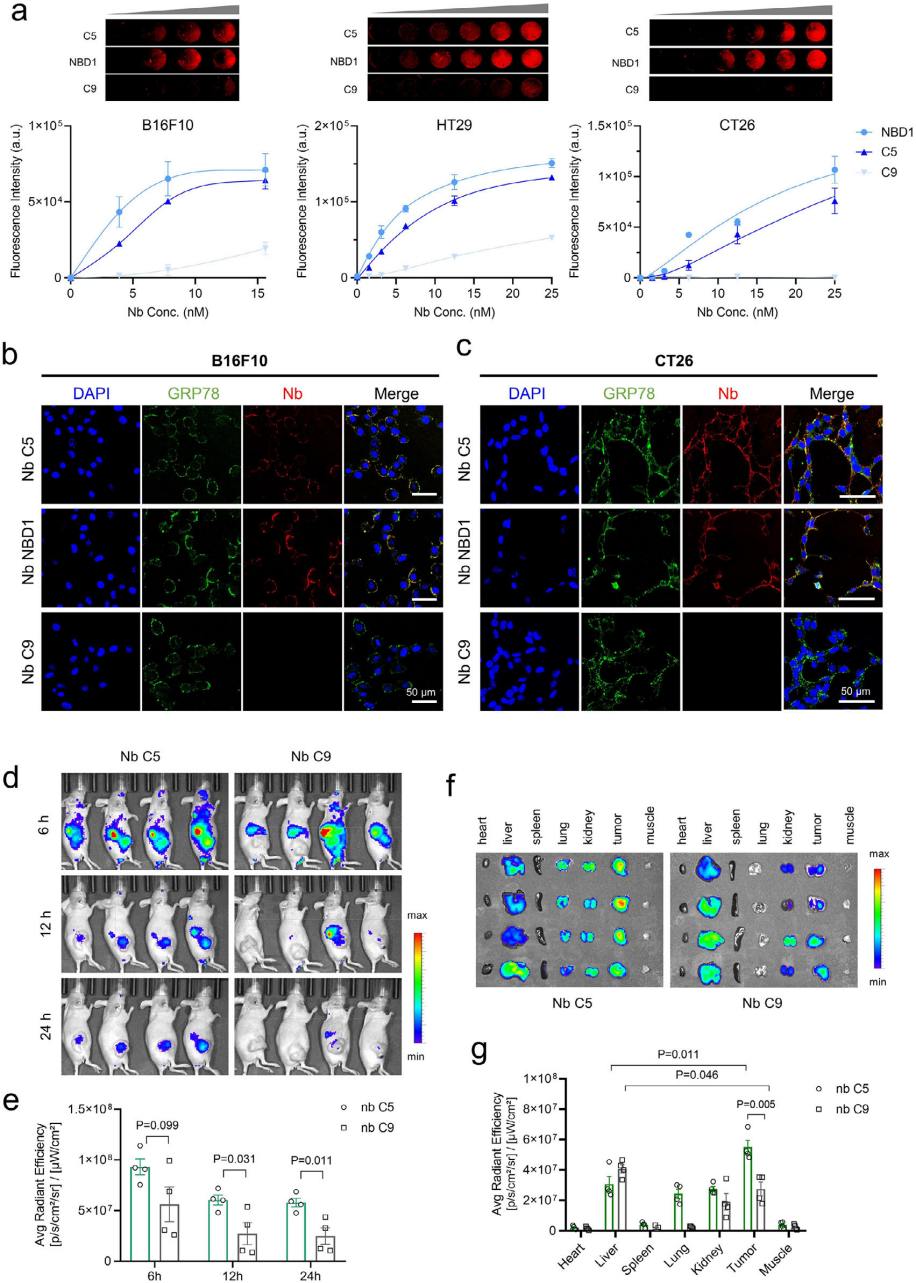
Nb C5 targets tumors with cell surface GRP78 (csGRP78) expression in vitro and in vivo. a) In‐Cell ELISA analysis detecting the binding of Nbs NBD1 and C5 to cancer cells with csGRP78 (mean ± SEM, n = 3 biological replicates). b,c) Immunofluorescence analysis showing co‐localization of GRP78 with GRP78‐targeted Nbs on the cell surface of B16F10 cells and CT26 cells. Scale bars, 50 µm. d,e) In vivo fluorescent imaging and quantification of tumor accumulation of Nb C5 and C9 in HT29‐bearing mice. Mice were intravenously injected with Cy5‐labeled Nbs (80 µg mouse^−1^) and imaged at different time points (mean ± SEM, n = 4 mice, unpaired two‐tailed Student's *t*‐test). f,g) Ex vivo imaging and quantification of biodistribution of Nbs in major organs and tumor tissues. The organs were collected for imaging 24 h after injection (mean ± SEM, n = 4 mice, two‐tailed unpaired Student's *t*‐test).

Having determined the specificity of the Nbs to the immobilized GRP78 protein, we next sought to evaluate their binding to cell‐derived GRP78. To this end, In‐cell ELISA and Nb immunofluorescence staining were performed using B16F10, HT29, or CT26 cell lines due to their high csGRP78 expression. In‐cell ELISA data revealed strong recognition of B16F10, HT29, and CT26 cells by Nb C5 and NBD1, compared to the control Nb C9 (**Figure** **3**a). Immunofluorescence staining further confirmed colocalization between Nb C5 and Nb NBD1 with GRP78 on the cell surface of B16F10 and CT26 cells (Figure 3b,c). To confirm the specificity of Nb C5 and Nb NBD1 binding to GRP78, a B16 cell pool knocked out GRP78 with a lentivirus‐based CRISPR‐Cas9 system was generated (Figure S8a, Supporting Information). Dot blot analysis revealed significantly reduced binding of Nbs NBD1 and C5 to GRP78 in the knockout cells compared to the control cells (Figure S8b, Supporting Information). Similarly, In‐cell ELISA demonstrated a marked decrease in the binding activity of both Nbs in the knockout cells (Figure S8c, Supporting Information). These findings collectively validate that Nbs NBD1 and C5 specifically interact with GRP78.

Next, we further investigated the in vivo targeting ability of the Nbs. Despite the high affinity of Nb NBD1, its poor stability under physiological conditions (37 °C) has hindered it's in vivo studies. To verify the in vivo targeting capability, Nb C5 and control C9 were labeled with Cy5 for fluorescence imaging. Fluorescence imaging of SDS‐PAGE gel demonstrated an efficient reaction of the probes with Nbs. (Figure S9, Supporting Information). HT29‐bearing mice were intravenously injected with Cy5‐labeled Nb C5 or Nb C9 (80 µg mouse^−1^) and imaged at different time points. The signal of Nb C5 in the tumors was significantly higher than in tumors receiving Nb C9 treatment over time after injection (Figure 3d,e). Notably, Nb C5 specifically accumulated in tumor tissues at 24 h post‐injection, with partial signal detected in the liver, kidney, and lung. Moreover, Nb C5 exhibited higher accumulation in the tumor than in the metabolic organ liver, unlike Nb C9, which showed similar levels in both organs (Figure 3f,g), highlighting the tumor‐targeting capability of Nb C5. These findings suggest that Nb C5 holds promise for delivering payloads for therapy and diagnostics against csGRP78‐expressing tumors.

### 2.4 The Anti‐GRP78 Immunotoxin, C5‐PE38, Targets csGRP78‐Expressing Tumor Cells and Induces Cell Death In Vitro

**Figure 4:**
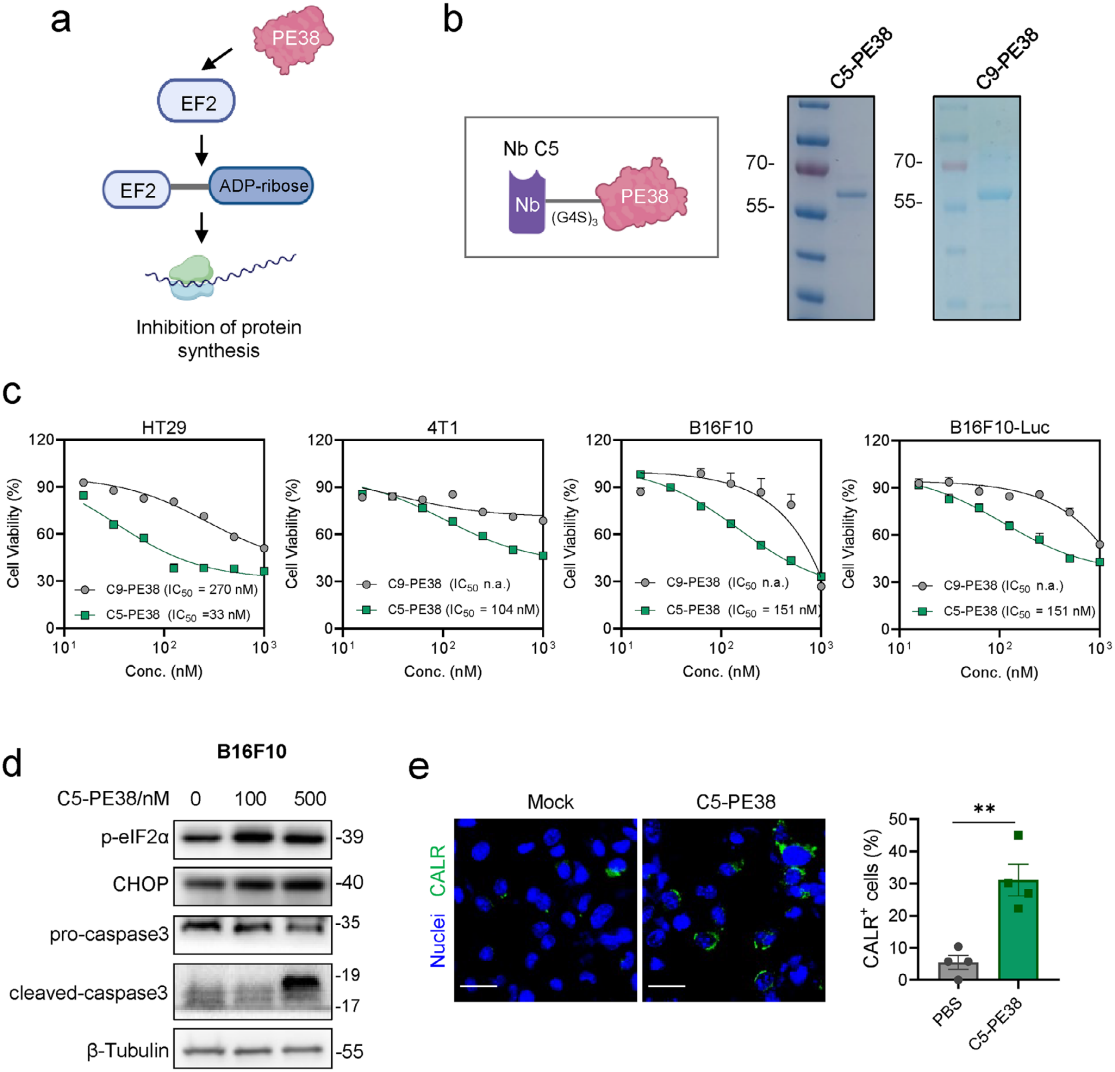
Nb C5‐derived PE38 immunotoxin displays enhanced cytotoxic activity toward target cells and induces apoptosis and immunogenic cell death. a) The mechanism of PE38‐based immunotoxins that induce adenosine diphosphate (ADP)‐ribosylation of elongation factor 2 (EF2) to halt protein synthesis and eventually lead to cell death. b) Construction and purification of GRP78‐targeted immunotoxins with Nb C5 and PE38. C5‐PE38 and the control immunotoxin C9‐PE38 were purified and identified by SDS‐PAGE followed by Coomassie staining. c) C5‐PE38 demonstrated higher cytotoxicity in tumor cells with csGRP78 expression. Cells were treated with C5‐PE38 or C9‐PE38 for 48 h and measured by CCK8 assay (n = 3–4 biological replicates). The IC_50_ values of C5‐PE38 are indicated in each graph. d) Immunoblot analysis of ER stress and apoptosis in B16F10 cells treated with C5‐PE38 (100 or 500 nM, 16 h). e) Representative immunofluorescence images of B16F10 cells showing increased CALR exposure after C5‐PE38 treatment (500 nM, 24 h; n = 4, unpaired two‐tailed Student's *t*‐test). Scale bars, 50 µm.

PE38 is a 38‐kDa truncated portion of PE, which induces ADP‐ribosylation of EF2 to halt protein synthesis and eventually lead to cell death^[^ ^21^ ^]^ (**Figure** **4**a). The GRP78‐targeted immunotoxin, C5‐PE38, was constructed by linking Nb C5 to PE38 using a G4S linker, while C9‐PE38 served as a control. SDS‐PAGE analysis confirmed the purification of both immunotoxins at the expected weights (≈55 kDa) with high purity (Figure 4b).

To assess the anti‐proliferative efficacy of GRP78‐targeted PE38, we conducted a CCK8‐based cytotoxicity assay of C5‐PE38 and C9‐PE38 on csGRP78‐expressing tumor cell lines. C5‐PE38 significantly reduced the viability of HT29, B16F10, B16F10‐Luc, and 4T1 cells with varied IC_50_ compared to the control C9‐PE38 (Figure 4c). However, C5‐PE38 showed low cytotoxicity in MC38 cells (Figure S10, Supporting Information), which express low levels of csGRP78, highlighting the targeting selectivity of C5‐PE38 for tumor cells. Furthermore, western blot analysis of B16F10 cells treated with C5‐PE38 revealed elevated levels of p‐eIF2α (phosphorylated eukaryotic translation initiation factor 2 alpha) and CHOP (C/EBP homologous protein), and cleaved‐caspase 3 (Figure 4d), which represent the activation of ER stress and apoptotic pathway, respectively.^[^ ^30^ ^]^ Cells undergoing ER stress may expose and release immunomodulatory damage‐associated molecular patterns (DAMPs) on their surface or into the extracellular space, promoting immunogenic cell death (ICD) and subsequent immune responses.^[^ ^31^ ^]^ PE immunotoxin has previously been shown to induce calreticulin (CALR) translocation and ATP release, key events during the ICD process.^[^ ^32^ ^]^ Consistent with this, we observed increased expression of CALR on the cell surface (Figure 4e). These results collectively demonstrate the in vitro antitumor activity of csGRP78‐targeted immunotoxin, implicating the promising potential for in vivo tumor suppression.

### 2.5 C5‐PE38 Exerts Potent Antitumor Activity in Various Tumor Models

**Figure 5:**
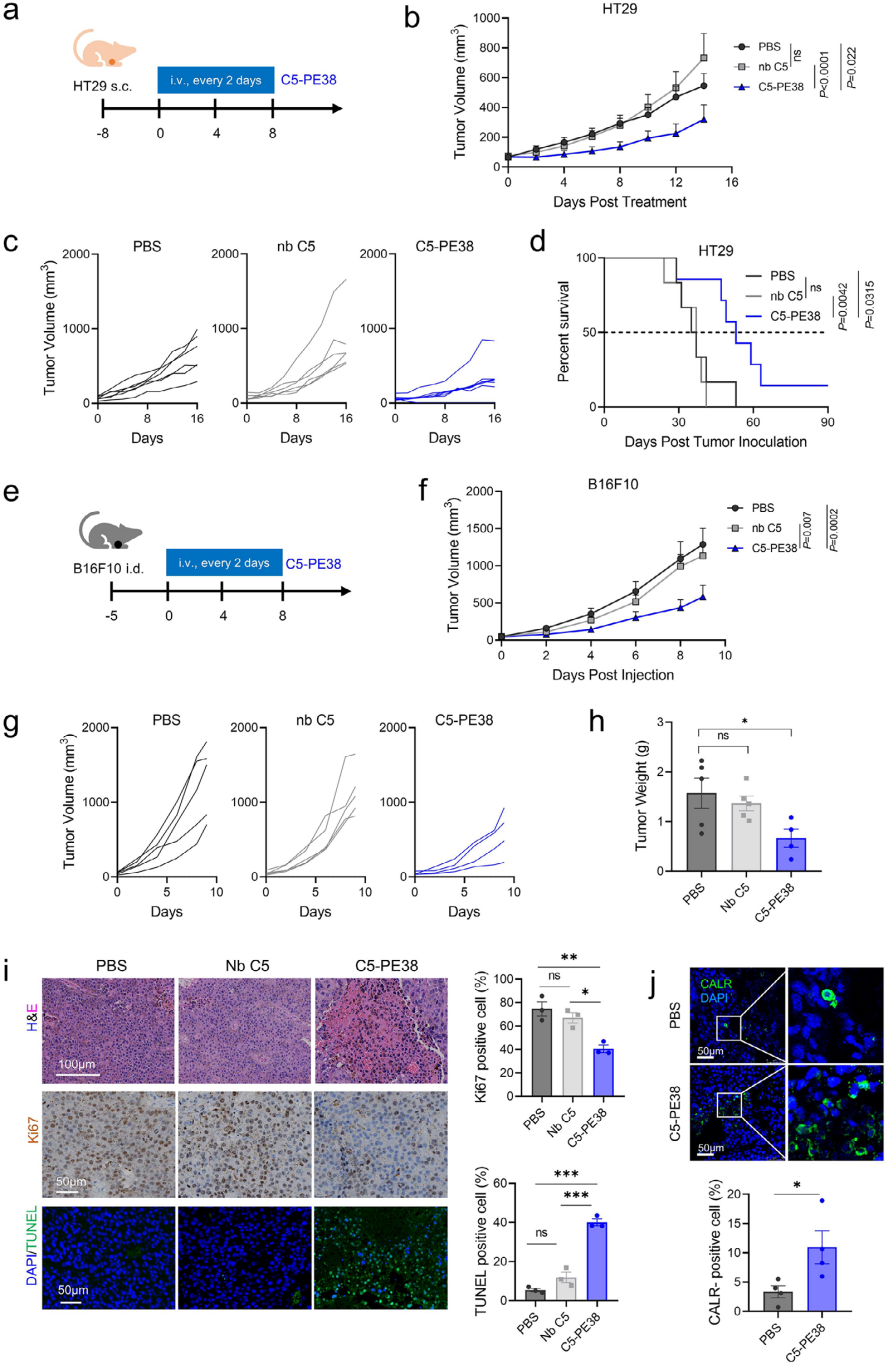
Anti‐GRP78 PE38 immunotoxin exerts potent antitumor effects against colorectal cancer and primary melanoma. a) Schematic of HT29 colorectal cancer model treated with PBS, Nb C5 (0.08 mg kg^−1^), or C5‐PE38 (0.3 mg kg^−1^) as indicated. BALB/c nude mice were injected subcutaneously with 5 × 10^6^ HT29 tumor cells. b–d) Tumor growth and Kaplan‐Meier survival analysis of HT29 tumor‐bearing mice (mean ± SEM, n = 6–7 mice per group). Mice were euthanatized as an endpoint when the tumor volume reached 1500 mm^3^ for survival analysis. e) Schematic of the B16F10 melanoma model treated with PBS, Nb C5, or C5‐PE38 as indicated. C57BL/6 mice were injected intradermally with 5 × 10^5^ B16F10 tumor cells. f,g) Tumor growth of B16F10 tumor‐bearing mice following treatment (mean ± SEM, n = 4–5 mice per group). h) Tumor weight from B16F10 tumor‐bearing mice on day 9 post‐treatment (mean ± SEM, n = 4–5 mice per group). i) Representative images and quantification of Ki67 and TUNEL staining in tumor sections from the B16F10 tumor model following different treatments (mean ± SEM, n = 3 mice per group). H&E staining was performed for histological analysis. Scale bars, 100 µm (H&E), 50 µm (Ki67 and TUNEL). j) Representative immunofluorescence images and quantification of CALR staining in tumor sections from the B16F10 tumor model (mean ± SEM, n = 4 mice per group). Scale bars, 50 µm. Ki67‐positive, TUNEL‐positive, and CALR‐positive cell frequencies were quantified using ImageJ software. Statistical significance was assessed by two‐way ANOVA with Tukey's multiple‐comparisons (b, f), log‐rank Mantel‐Cox test (d), one‐way ANOVA with Tukey's multiple‐comparisons (h, i), and unpaired Student's *t*‐test (j). *p < 0.05, **p < 0.01, ***p < 0.001, and ****p < 0.0001.

To interrogate the in vivo therapeutic benefit and safety of anti‐GRP78 immunotoxin, we tested C5‐PE38 against two xenograft models expressing csGRP78. We first investigated its anti‐tumor potency in a subcutaneous colorectal cancer model induced by HT29 cells. On day ‐8, HT29 cells (5 × 10^6^) were implanted subcutaneously, and 8 days later, mice were intravenously administered 0.3 mg kg^−1^ of C5‐PE38 or 0.08 mg kg^−1^ of Nb C5 (equimolar dose of C5‐PE38) every 2 days for five injections (**Figure** **5**a). The control C9‐PE38 was excluded due to its lethal toxicity to mice, as determined in our previous work and other reports.^[^ ^33^ ^]^ As expected, C5‐PE38 exhibited significant tumor‐inhibitory effects, whereas C5 exhibited no discernible effects (Figure 5b,c). The median survival time of mice treated with C5‐PE38 was extended by 17 days compared to the PBS control group (Figure 5d). Subsequently, we assessed the efficacy of C5‐PE38 in a melanoma model induced by B16F10 cells. 5 × 10^5^ B16F10 cells were implanted intradermally to establish the melanoma model. 5 days later, mice received a similar treatment regimen as the HT29 model (Figure 5e). Similarly, mice treated with C5‐PE38 showed delayed tumor growth compared to control groups (Figure 5f–h).

To validate the therapeutic efficacy of C5‐PE38, we examined its impact on B16F10 tumor tissues by histological analysis. Compared to controls, C5‐PE38‐treated tumors showed increased TdT‐mediated dUTP‐biotin nick end labeling (TUNEL)‐positive cells, indicating enhanced cell apoptosis, alongside increased noticeable cell death in H&E staining (Figure 5i). In addition, C5‐PE38 was found to inhibit tumor proliferation through Ki67 staining, corroborating the antitumor effect of C5‐PE38 (Figure 5i). Importantly, we observed an increase in CALR‐positive cells in tumors treated with C5‐PE38 (Figure 5j), suggesting the induction of ICD after C5‐PE38 treatment.

We also evaluated the safety of the immunotoxin throughout the entire experimental period. There were no significant changes in animal weight in either xenograft model and no statistically significant difference was found among all the groups (Figure S11a,b, Supporting Information). Blood biochemical indexes revealed no abnormalities and pathology analysis of vital organs post‐C5‐PE38 treatment showed no obvious damage (Figure S11c–e, Supporting Information), indicating that anti‐GRP78 PE38 has an excellent safety profile.

### 2.6 C5‐PE38 Induces STING‐Dependent Type I IFN Response in the Tumor Microenvironment

**Figure 6:**
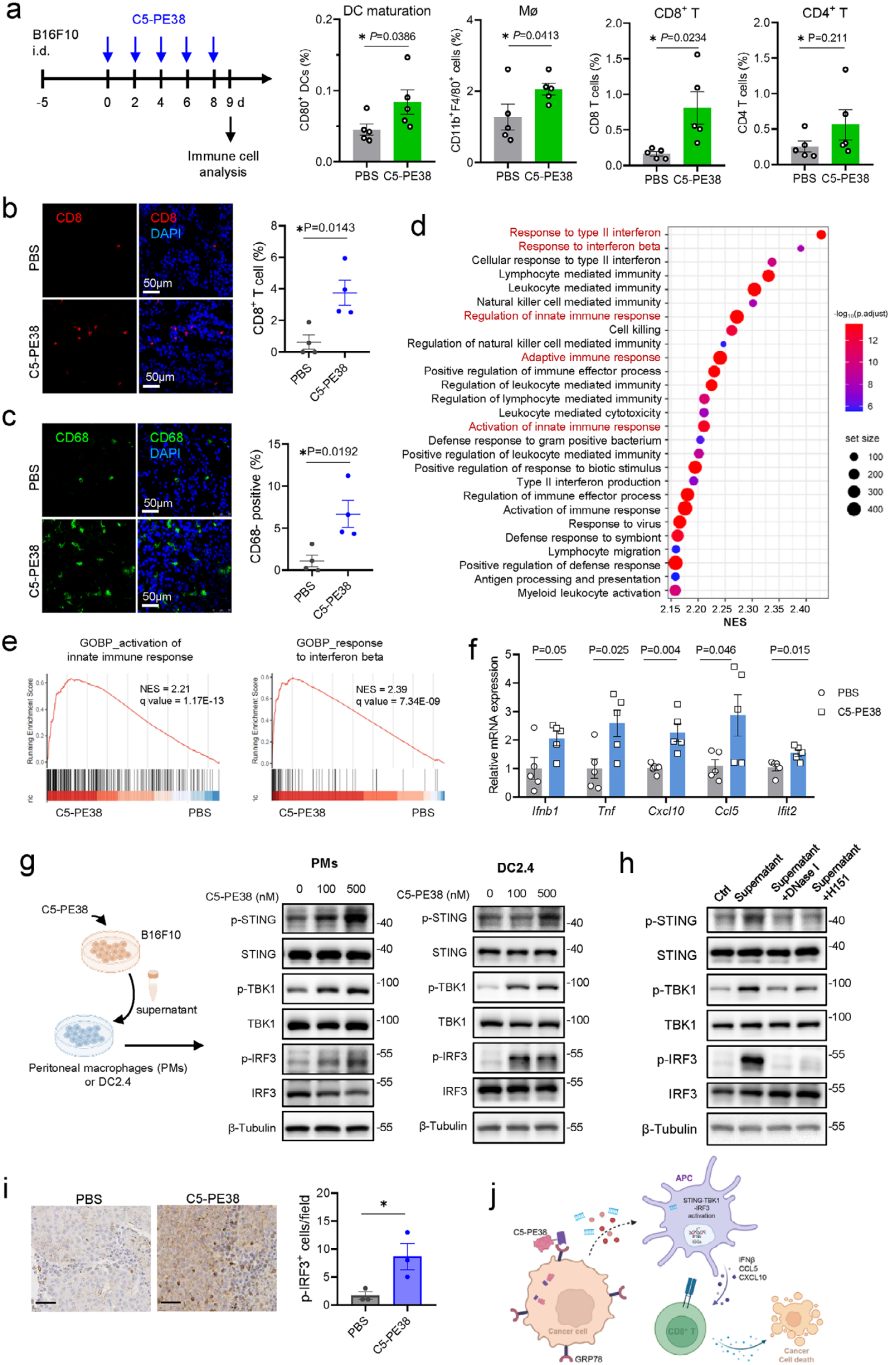
C5‐PE38 induces STING‐dependent type I IFN response in the tumor microenvironment. a) Flow cytometry analysis of DC maturation (CD11c^+^CD80^+^), macrophages (CD11b^+^F4/80^+^), CD8^+^ T cells (CD3^+^CD4^−^CD8^+^) and CD4^+^ T cells (CD3^+^CD4^+^CD8^−^) in tumor tissues from mice treated with PBS or C5‐PE38 (mean ± SEM, n = 5 mice per group). Tumors were collected after five doses of treatment. b,c) Representative immunofluorescence images and quantitative analysis of macrophage and CD8^+^ T cell infiltration in tumor tissues (mean ± SEM, n = 4). Scale bar, 50 µm. d) Gene Set Enrichment Analysis (GSEA) of RNA‐seq data to identify differentially expressed gene sets in tumors treated with C5‐PE38 compared to PBS‐treated tumors using the gene sets derived from GOBP in MSigDB. B16F10 tumor tissues were collected after five doses of treatment. The bubble plot displays the top enriched pathways based on the normalized enrichment score (NES) comparing C5‐PE38 versus PBS‐treated groups (n = 4 per group). e) GSEA results show that biological process pathways associated with the activation of innate immune response and response to interferon beta are significantly enriched in tumors treated with C5‐PE38. An adjusted *p*‐value of <0.05 was considered statistically significant. f) qPCR analysis of mRNA expression levels of Ifnb1, Tnf, and representative interferon‐stimulated genes (ISGs) in B16F10 tumor tissue (mean ± SEM, n = 5). Actin was used as an internal control. g) Western blot analysis evaluating the STING pathway in peritoneal macrophages and DC2.4 cells after 4‐h stimulation with cell supernatants (n = 3). The cell supernatant was collected from B16F10 cells treated with C5‐PE38 for 24 h. h) Examination of the STING pathway in DC2.4 cells after 4‐h stimulation with cell supernatants containing different inhibitors (n = 3). The supernatants were collected from B16F10 cells treated with C5‐PE38 for 24 h. To degrade DNA, 2 U mL^−1^ DNase I was added to the supernatant and incubated at 37 °C for 2 h. To suppress the STING pathway, DC2.4 cells were pre‐treated with H151 (5 µM) for 4 h. i) Representative immunohistochemistry images and quantification of p‐IRF3 staining in tumor tissues from the B16F10 tumor model (mean ± SEM, n = 3 mice per group). Scale bars, 50 µm. j) Mechanism illustrating STING‐TBK1‐IRF3 pathway activation in antigen‐presenting cells via tumor‐derived nucleic acids upon C5‐PE38 treatment. Statistical significance was assessed by unpaired Student's *t*‐test (a, b, c, f, i). *p < 0.05, **p < 0.01, ***p < 0.001, and ****p < 0.0001.

Next, we aimed to decipher the mechanism underlying the anti‐tumor effect of C5‐PE38. Past clinical observations and animal studies suggest that the activation of the immune system plays a crucial role in the anti‐tumor effects of PE immunotoxins.^[^ ^21^, ^24^, ^26^ ^]^ Therefore, tumor‐infiltrating immune cell subsets were examined using flow cytometry (Figure S12, Supporting Information). The analysis demonstrated that C5‐PE38 treatment increased the population of CD8^+^ T cells while did not alter the population of CD4^+^ T cells relative to PBS treatment (**Figure** **6**a). Further investigation into innate immune cells indicated that C5‐PE38 treatment promoted macrophage infiltration and dendritic cells (DC) maturation while maintaining the DC population. The immunofluorescent analysis further confirmed the infiltration of CD8^+^ T cells and macrophages (Figure 6b,c). Next, we isolated B16F10 tumors receiving C5‐PE38 or PBS treatment and performed bulk RNA‐seq analysis. C5‐PE38 induced striking changes in gene expression and gene set enrichment analysis (GSEA) revealed a prominent immune activation signature (Figure 6d, Figure S13, Supporting Information). Notably, pathways related to interferon (IFN) signaling, and innate and adaptive immune responses were significantly enriched after C5‐PE38 treatment (Figure 6d). Importantly, the top 15 GOBP enriched in the C5‐PE38 group included the response to IFN‐β, regulation and activation of innate immune response (Figure 6d,e). Type I IFNs prompt the autocrine or paracrine release of cytokines and chemokines that modulate innate and adaptive immune responses.^[^ ^34^ ^]^ Given the role of type I IFN signaling in antitumor immunity,^[^ ^34^ ^]^ we investigated whether the immune cell alterations induced by C5‐PE38 were mediated by IFN‐β signaling. QPCR analysis of tumor samples revealed elevated expression of Ifnb and Tnf, a pro‐inflammatory cytokine, after C5‐PE38 treatment (Figure 6f). Additionally, several IFN‐stimulated genes (ISGs) were upregulated, including Ccl5 and Cxcl10, which are essential chemokines for attracting tumor‐infiltrating lymphocytes (TILs),^[^ ^31^ ^]^ indicating activation of the type I IFN pathway.

Cytoplasmic dsDNA, one of the crucial stimulators of innate immunity, can evoke type I IFN production through the cyclic GMP‐AMP synthase (cGAS)‐STING pathway.^[^ ^31^, ^35^ ^]^ As PE38 induces apoptosis and ICD of tumor cells, we hypothesized that cytosolic DNA released from C5‐PE38‐treated cells may activate the STING pathway in antigen‐presenting cells (APCs). After treating B16F10 cells with C5‐PE38, we observed an increase in the nucleic acid content in the supernatant (Figure S14, Supporting Information). Indeed, stimulation of mouse peritoneal macrophages and DC2.4 cells with the supernatants from C5‐PE38‐treated B16F10 cells elevated expression of p‐STING and downstream phospho‐TANK binding kinase 1 (p‐TBK1) and phospho‐interferon regulatory factor 3 (p‐IRF3) signaling (Figure 6g, Figure S15a, Supporting Information), indicating activation of the STING‐TBK1‐IRF3 axis. Meanwhile, we did not observe activation of the STING‐TBK1‐IRF3 axis in isolated primary natural killer (NK) cells under the same conditions (Figure S16, Supporting Information). This suggests that C5‐PE38 does not activate the STING pathway in NK cells under the experimental conditions tested. This could be due to differences in sensitivity to dsDNA among macrophages, DCs, and NK cells, or possibly an insufficient amount of dsDNA in the supernatants of C5‐PE38‐treated cancer cells. Further investigation is needed to elucidate the exact mechanism. Furthermore, pretreating the supernatant with DNase or co‐culturing the DC2.4 cells with H151 (a STING antagonist) suppressed the activation of the STING pathway (Figure 6h, Figure S15b, Supporting Information). This suggests that dsDNA released from tumor cells treated with C5‐PE38 activates the STING pathway in APCs. Upon phosphorylation, IRF3 translocates to the nucleus, where it activates the transcription of type 1 IFNs and ISGs, which is a hallmark of activation of the STING pathway.^[^ ^31^ ^]^ Our immunohistochemical analysis of tumor tissues demonstrated elevated nuclear IRF3 expression in the C5‐PE38 treatment group (Figure 6i), indicating that the downstream of STING pathway was initiated. To further investigate the activation of this pathway, we assessed the protein levels of IFN‐β in the culture supernatants of peritoneal macrophages and DC2.4 cells, as well as in the supernatants from B16F10 tumor cells. Our results showed that C5‐PE38 supernatant significantly induced the secretion of IFN‐β in both peritoneal macrophages and DC2.4 cells, but no such induction was observed in B16F10 tumor cells (Figure S17a, Supporting Information). This suggests that C5‐PE38 treatment transactivates the STING pathway in APCs like macrophages and DCs, leading to type I IFN production, but does not directly induce IFN‐β secretion in tumor cells. A significant TNF‐α production was also observed in peritoneal macrophages and DC2.4 cells following stimulation (Figure S17b, Supporting Information). Furthermore, we conducted immunofluorescence analysis on tumor tissues and observed a clear increase in IFN‐β expression within the TME following C5‐PE38 treatment (Figure S18, Supporting Information). These results collectively support the hypothesis that STING‐mediated type I IFN signaling is upregulated in response to C5‐PE38 treatment and contributes to the reprogramming of TME.

In summary, these findings suggest that C5‐PE38 treatment triggers a STING‐mediated type I IFN response in APCs via released nucleic acids, potentially reshaping the tumor microenvironment and eliciting CD8^+^ T cell‐mediated antitumor immunity (Figure 6j).

### 2.7 C5‐PE38 and α‐PD1 Combination Elicits Robust Systematic Immunity against Primary and Metastatic Melanoma

**Figure 7:**
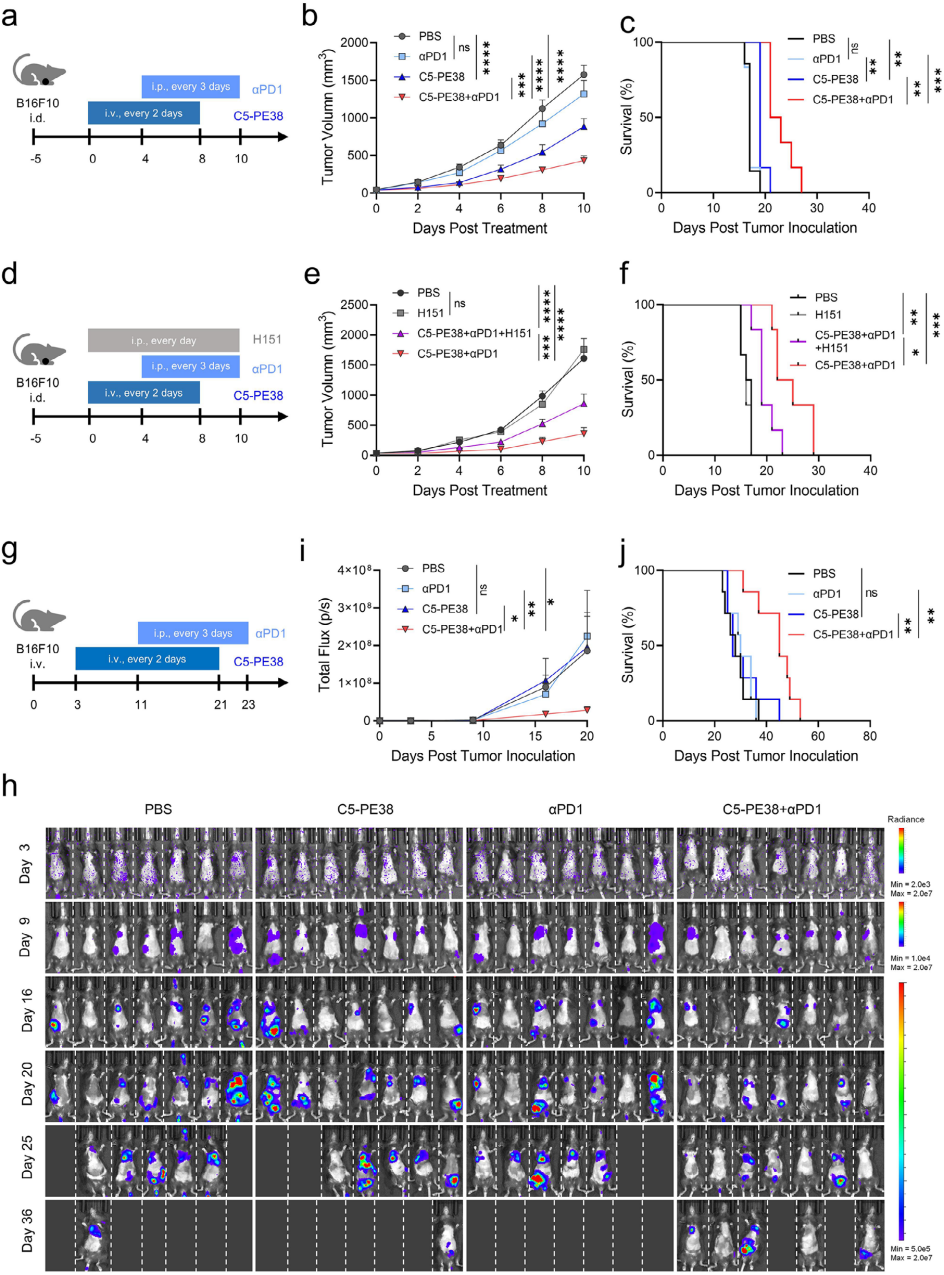
Anti‐GRP78 PE38 immunotoxin and α‐PD1 combination elicits robust systematic immunity against primary and metastatic melanomas. a) Schematic illustrating the experimental design for the evaluation of the antitumor performance of C5‐PE38 and α‐PD1 combination against B16F10 primary melanoma. C57BL/6 mice were injected intradermally with 5 × 10^5^ B16F10 tumor cells. Mice were treated with PBS, C5‐PE38, α‐PD1, or C5‐PE38 with α‐PD1 as indicated. b,c) Tumor growth and Kaplan‐Meier survival analysis of B16F10 tumor‐bearing mice (mean ± SEM, n = 6–7 mice per group). Mice were euthanatized as an endpoint when the tumor volume reached 2000 mm^3^ for survival analysis. d) Schematic illustrating the experimental design for evaluating the impact of STING inhibition on the antitumor performance of C5‐PE38 and α‐PD1 combination against B16F10 primary melanoma. Mice were treated as indicated. e,f) Tumor growth and Kaplan‐Meier survival analysis of B16F10 tumor‐bearing mice under the STING inhibition combined with various treatments (mean ± SEM, n = 6 mice per group). Mouse survival was similarly assessed as mentioned above in (c). g) Schematic illustrating the experimental design for evaluating the anti‐metastasis potential of C5‐PE38 and α‐PD1 combination against B16F10‐Luc melanoma metastasis. The metastatic melanoma model was generated by injecting B16F10‐Luc cells (2.5 × 10^5^ cells) into C57BL6 mice intravenously. From day 3, mice were treated with PBS, C5‐PE38, α‐PD1, or C5‐PE38 with α‐PD1 as indicated. h) Serial in vivo bioluminescence imaging of B16F10‐Luc‐bearing mice indicating the whole metastatic lesion in mice. The bioluminescence of mice was measured using an IVIS Lumina imaging system after intraperitoneal administration of D‐luciferin substrate (150 mg kg^−1^). i) Quantitative assessment of whole‐body bioluminescence in mice (mean ± SEM, n = 7 mice per group). j) Kaplan‐Meier survival analysis of B16F10‐Luc‐bearing mice. Statistical significance was assessed by two‐way ANOVA with Tukey's multiple‐comparisons (b, e, i) and log‐rank Mantel‐Cox test (c, f, j). *p < 0.05, **p < 0.01, ***p < 0.001, and ****p < 0.0001.

The presence of CD8^+^ cytotoxic T cells in tumors contributes to potent antitumor immunity. However, the immunosuppressive TME induces T cell dysfunction and exhaustion through the expression of the PD‐1 inhibitory receptor. In our study, we observed that in the B16F10 tumor model, a large proportion of CD3^+^ T cells expressed PD‐1, regardless of C5‐PE38 treatment (Figure S19, Supporting Information). Therefore, we next explored whether the addition of anti‐PD‐1 antibody (α‐PD1), a well‐known T‐cell immune checkpoint blocker, could synergistically enhance the tumor‐suppressing effects of C5‐PE38 in mice with locally primary and metastatic melanomas. Impressively, the combination of α‐PD1 and C5‐PE38 not only suppressed tumor proliferation but also significantly prolonged the survival of mice compared to C5‐PE38 monotherapy (**Figure** **7**a–c, Figure S20a, Supporting Information). To confirm whether STING activation in tumors is essential for C5‐PE38 plus α‐PD1‐mediated antitumor efficacy, we inhibited the host STING pathway using H151, through daily intraperitoneal injections to the B16F10‐bearing mice (Figure 7d). Injection of H151 alone had no effect on tumor growth, however, it significantly attenuated the therapeutic efficacy of C5‐PE38 plus α‐PD1 treatment (Figure 7e,f, Figure S20b, Supporting Information). These findings emphasize the essential role of STING activation in C5‐PE38 plus α‐PD1‐mediated antitumor immunity. Furthermore, we investigated whether the combination therapy of C5‐PE38 and α‐PD1 is effective against metastatic melanoma. In the metastatic melanoma model, the intravenous injection of B16F10‐Luc cells primarily resulted in lung metastasis, with additional spread to regions such as the abdomen and legs, underscoring the aggressiveness of this model. Despite its effectiveness against primary melanomas, C5‐PE38 alone failed to hinder tumor metastasis and improve survival rates (Figure 7g–j), probably due to its high aggressiveness. Similarly, α‐PD1 monotherapy exhibited no response to both primary tumors and metastases (Figure 7b,c,i,j). However, the combination of α‐PD1 and C5‐PE38 exhibited potent therapeutic effectiveness and significantly prolonged the survival of mice compared to C5‐PE38 or α‐PD1 monotherapy (Figure 7h–j). In addition, this combined therapy did not induce weight loss in both models (Figure S21, Supporting Information), indicating its favorable biocompatibility and potential for clinical translation. Due to the location and depth of the metastases, as well as interference from the mouse's fur, bioluminescence signals were undetectable in some mice despite signs of disease progression. For example, Mouse 3 in the C5‐PE38 group began showing weight loss and limping from day 23, suggestive of bone metastases, even though no strong bioluminescent signal was observed. Similarly, Mouse 6 in the α‐PD‐1 group exhibited weight loss without detectable tumors (Figure S21c, Supporting Information). Collectively, these results demonstrate that csGRP78 nanobody‐directed immunotoxin not only inhibits tumor cell proliferation but also greatly reprograms tumor microenvironment via STING activation in APCs, which may entail the adaptive antitumor immunity and expedite the efficacy of immunotherapy.

## 3 Discussion

In this study, we designed and validated a GRP78 Nb‐based system for targeting solid tumors, with a focus on melanoma and colorectal cancer. First, we identified a panel of GRP78‐binding Nbs through phage library screening and subsequent affinity characterization. Next, we developed a GRP78‐targeting PE38 immunotoxin to showcase the therapeutic potential of this target. Our findings suggest that, in addition to eliminating tumor cells in a targeted manner, C5‐PE38 also indirectly stimulates STING‐mediated T‐cell antitumor immunity through DNA released from dying tumor cells. Furthermore, its combination with anti‐PD1 antibody elicited robust antitumor immunity against primary and metastatic melanoma and prolonged mice survival. This study contributes to bridging the gap in understanding the anti‐tumor immune mechanisms of PE‐based immunotoxins.

ER stress‐induced translocated proteins such as GRP78 may represent a potential target for pan‐cancer types. Through tissue microarray analysis, we found that multiple cancer types exhibit expression of csGRP78, without significant tumor type preference. This suggests that targeted therapy against csGRP78 may be applicable to multiple tumor types. Nbs, with their high affinity, good tissue penetration, and cost‐effectiveness, have demonstrated broad applicability in cancer therapy, viral pathogen neutralization, and diagnostic detection.^[^ ^36^ ^]^ To obtain high‐affinity ligands for targeting GRP78, we conducted Nb screening using a phage display library against three fragments of GRP78. Among the potential positive clones identified, three Nbs C5, NBD1, and NGS3, were found to bind to both human and murine GRP78, with C5 showing the most favorable results upon comprehensive evaluation. A previous study reported a GRP78‐targeting Nb with an affinity of only 2.1 × 10^−7^ M; however, it lacked binding identification for murine GRP78 and in vivo data.^[^ ^37^ ^]^ In contrast, our identified Nb C5 demonstrated a higher affinity (9.2 × 10^−8^ M) and exhibited excellent tumor‐targeting efficacy in both cellular and animal models.

The resultant C5‐PE38 immunotoxin possesses several critical attributes that enhance its efficacy in cancer therapy, while concurrently ensuring its safety in vivo. First, the high surface translocation of GRP78 in cancer cells, with minimal translocation in normal tissues, mitigates on‐target off‐tumor toxicity against healthy organs. This has been corroborated through histopathological examination of major organs and blood biochemical indexes. Furthermore, the extended CDR3 of the Nb C5 enables access to the concealed antigen epitope, and its high affinity (9.2 × 10^−8^ M) ensures high specificity to target cells. Additionally, the unique properties of Nbs, including their small size (4 nm in length, 2.5 nm in diameter) and deep tissue penetration, facilitate superior tumor penetration of the immunotoxin compared to scFv‐based PE38.^[^ ^38^ ^]^ Moreover, the absence of the fragment crystallizable region (Fc) avoids nonspecific cellular binding to certain immune cells. The toxin PE construct has also undergone modification to exclude the original receptor binding domain Ia and partial domain Ib, preventing nonspecific recognition and improving cellular tolerance.^[^ ^39^ ^]^

Past clinical observations and animal studies suggest that the activation of the immune system plays a crucial role in the anti‐tumor effects of PE immunotoxins,^[^ ^20^, ^23^, ^24^, ^26^ ^]^ however, the specific mechanisms remain unclear. In our study, the RNA‐seq analysis of C5‐PE38‐treated B16F10 tumor tissue revealed activation of innate and adaptive immune responses, with significant enrichment of response to IFNβ pathways. Given that the cGAS‐STING signaling pathway serves as a key regulator bridging innate and adaptive immunity,^[^ ^34^ ^]^ we speculate that C5‐PE38 treatment may activate the STING pathway in the TME. Studies have shown that the STING pathway is essential for T‐cell responses induced by radiation and certain chemotherapeutics.^[^ ^31^ ^]^ In mammalian cells, cytosolic DNA can be detected by the DNA sensor cGAS, which generates the second messenger cGAMP to activate the innate immune adapter STING, which, in turn, leads to TBK1‐IRF3 signaling and transcription of type I IFN genes.^[^ ^31^ ^]^ Type I IFN response is crucial for activating antitumor immunity and recruiting immune cells in TME.^[^ ^40^ ^]^ Our flow cytometry analysis confirmed an increase in mature DCs resident in the TME, as well as infiltration of macrophages and CD8^+^ T cells. The latter can be explained by the upregulated expression of Ccl5 and Cxcl10, classical downstream genes of STING and ICD induction in cancer cells by C5‐PE38, both of which facilitate the recruitment of CD8^+^ T cells, NK cells, and monocytes to tumor sites.^[^ ^41^ ^]^ Elevated expression of type I IFNs or ISGs within tumors is linked to favorable disease outcomes across several cancer types.^[^ ^34^ ^]^ Excessive DNA fragments produced in the nucleus or mitochondria due to external stimuli like chemotherapy, radiotherapy, and targeted therapy can leak into the cytoplasm or extracellular space, where they can be engulfed by APCs and sensed by intracellular cGAS.^[^ ^31^ ^]^ It should be noted that the generated dsDNA fragments can be transferred between cells through various mechanisms, such as extracellular vesicles, membrane fusion, phagocytosis, and forming complexes with specific proteins, thereby activating the cGAS‐STING signaling pathway in neighboring cells.^[^ ^42^ ^]^ To further confirm our hypothesis, we stimulated peritoneal macrophages and DC2.4 cells with supernatants from C5‐PE38‐treated tumor cells, which resulted in activation of the STING‐TBK1‐IRF3 signaling axis in both cell types. Importantly, the activation of the STING pathway was abrogated by DNase treatment or in the presence of H151, suggesting that the dsDNA derived from C5‐PE38‐induced cell death is the key mediator. Previous studies have found that PE immunotoxins induce ER stress and eIF2 phosphorylation,^[^ ^43^ ^]^ as well as promote ICD.^[^ ^32^, ^44^ ^]^ Consistently, we observed elevated surface translocation of CALR following C5‐PE38 treatment in vitro and in vivo, suggesting the occurrence of ICD. During ICD, dying tumor cells also release or expose nucleic acids, which act as an immunostimulatory DAMP.^[^ ^45^ ^]^ The extracellular dsDNA likely engages the cGAS‐STING pathway in APCs, thereby triggering an immune response. It has been demonstrated that the released cytosolic dsDNA from dying cells resulted from UV irradiation could significantly transactivate STING signal in APCs in TME,^[^ ^46^ ^]^ which aligns with our results. Therefore, the occurrence of C5‐PE38‐induced ICD, similar to the typical ICD inducer UV irradiation,^[^ ^47^ ^]^ is particularly crucial to indirectly promote the activation of the STING pathway.

Immune checkpoint therapies targeting inhibitory receptors on T cells have revolutionized the treatment of melanoma by overcoming tumor immune suppression.^[^ ^48^ ^]^ However, their efficacy is limited to a subset of patients with pre‐existing CD8^+^ T cells in the tumor.^[^ ^49^ ^]^ We, therefore, investigated if the antitumor efficacy of C5‐PE38 can be augmented with PD‐1 blockade. Our study in an immunocompetent murine primary melanoma model revealed that this combination therapy elicited targeted tumor cell cytotoxicity and an effective adaptive T‐cell response, leading to prolonged survival. These findings are in agreement with a previous study showing that LMB‐100 combined with anti‐PD‐1 antibody increases antitumor efficacy in a 531LN2‐hMSLN syngeneic mouse model, which attributed to increased CD8^+^ T cells in the TME.^[^ ^26^ ^]^ Inspired by the promising outcome of this combination therapy, we further examined their efficacy in a metastatic model induced by intravenous injection of B1F10‐Luc cells. Unexpectedly, C5‐PE38 alone did not show any improvement in metastatic inhibition or survival compared to the PBS group, which may be ascribed to the aggressive nature of this model. However, despite this, the addition of anti‐PD1 antibody to C5‐PE38 significantly slowed down tumor metastasis progression and extended survival compared to both monotherapies (P < 0.01). These findings highlight the potential of C5‐PE38 to modulate the TME and sensitize tumors to PD1 blockade. Notably, the introduction of H151, a STING antagonist, partially attenuated the anti‐tumor activity of C5‐PE38 combined with an anti‐PD1 antibody in a B16F10 model. Given the inherent toxicity of C5‐PE38 inhibiting protein synthesis in cancer cells, H151 did not completely reverse the effects of the combination therapy. It is worth noting that PD‐1 blockade alone did not exhibit significant therapeutic effects in this model. Activation of the STING pathway has been shown to enhance the efficacy of immune checkpoint blockade.^[^ ^50^ ^]^ In our study, the activation of the STING pathway via C5‐PE38 was shown to enhance the anti‐tumor immune response, likely by promoting the infiltration of T cells and the overall reprogramming of the TME. When the STING pathway was inhibited by H151, the therapeutic efficacy of the combination treatment was weakened, suggesting that the STING pathway mediates the synergistic effects between C5‐PE38 and α‐PD‐1. Therefore, these findings suggest that the combination of C5‐PE38 and α‐PD1 relies on the STING pathway for optimal therapeutic effect.

Our study still has some shortcomings and areas for improvement. Additional tumor models, particularly immunocompetent models, should be employed to confirm the efficacy of C5‐PE38 in csGRP78‐expressing cancers. What's more, further investigations are still needed to identify the specific non‐malignant cells in the TME responsible for STING activation and to elucidate the specific signals transferred from tumor cells to these cells. Additionally, exploring the potential combination of C5‐PE38 with other immunotherapies, such as adoptive cell transfer and cancer vaccines, represents a promising avenue for future research.

In sum, our study not only emphasizes the promising potential of csGRP78 as a universal target for cancer therapy but also introduces a GRP78‐targeting immunotoxin as a potent agent against csGRP78‐expressing tumors. Considering the common occurrence of GRP78 translocation on cancer cell surfaces following various therapies like radiotherapy and chemotherapy, the GRP78‐targeted immunotoxin holds promise as a combinational approach for these therapies. Furthermore, our findings provide novel insights into the mechanism by revealing that PE immunotoxins induce STING‐mediated IFN‐I signaling within antigen‐presenting cells, thereby eliciting CD8 T cell‐mediated antitumor immunity. These findings significantly advance our understanding and clinical application of PE immunotoxin therapy.

## 4 Experimental Section

### Pan‐Cancer Analysis of HSPA5 (GRP78) using TCGA and CPTAC Datasets

For the pan‐cancer analysis of HSPA5, this work utilized Gepia2 (http://gepia2.cancer‐pku.cn/)^[^ ^51^ ^]^ to analyze RNA‐seq data from TCGA and the GTEx and utilized cProSite (https://cprosite.ccr.cancer.gov/)^[^ ^52^ ^]^ to analyze proteomic data from the CPTAC. This allowed comparing the abundance of HSPA5 protein between tumor tissues and their corresponding normal tissues. HSPA5 expression levels were extracted from both datasets. The RNA‐seq data were TPM normalized and log‐transformed. Differential expression between tumor and normal tissues was examined using the T‐test. UALCAN (https://ualcan.path.uab.edu/)^[^ ^53^ ^]^ was employed to track changes in protein abundance across cancer progression stages in CPTAC data. Survival analysis was conducted using Kaplan‐Meier curves and Cox regression to evaluate the association between HSPA5 expression and patient survival, facilitated by Gepia2. All statistical analyses were performed using R and web‐based interfaces, with a significance threshold set at *p* < 0.05.

### Nanobody Screening

GRP78‐binding Nb screening was performed using a naïve alpaca Nb phage display library with a diversity of 2 × 10^9^, following methods outlined in the previous study.^[^ ^38b^ ^]^ In brief, three antigens (hGRP78, NBD domain, SBD domain) were coated in immunotubes separately. After three rounds of panning against each antigen, 192 individual clones were selected for phage enzyme‐linked immunosorbent assay (ELISA) identification. Phage clones exhibiting a more than threefold increase in OD450 value (antigen versus BSA) were considered potentially positive clones and subjected to sequencing. Additionally, the amplified third‐round phage eluate from three antigens was sent for deep sequencing, and Nbs with sequence frequencies ≥ 0.9% were chosen to assess the binding ability. Finally, a total of 11 independent Nb sequences were obtained through the screening of all three antigens.

### Nanobody Purification

To purify and identify the Nbs, the coding region of each Nb was cloned into the pET‐14b vector with an HA tag at the C‐terminal and His tag at the N‐terminal. A cysteine amino acid was attached at the end of Nb for subsequent imaging probe labeling. The recombinant plasmids were then introduced into BL21 (DE3) competent cells and expressed. Induction was carried out with 0.3 mM IPTG at 16 °C overnight. Cell pellets were harvested and high‐pressure lysed in a lysis buffer. Supernatants were obtained via centrifugation (12 000 g, 45 min) and subjected to Ni‐NTA purification. Furthermore, the protein eluate was further purified using an AKTA Pure System (GE Healthcare, USA). Identification of the purified proteins was performed through SDS‐PAGE and Coomassie Blue staining.

### ELISA

The antigens (hGRP78 or mGRP78, 10 µg mL^−1^) were immobilized on ELISA plates overnight at 4 °C. Subsequently, the plates were blocked with 3% BSA/PBS for 1 h at room temperature (RT) to prevent nonspecific binding. Serially diluted solutions of HA‐tagged Nbs were then added and incubated at RT for 1 h to allow for specific binding to the immobilized antigens. Following three washes with PBST to remove unbound Nbs, the plates were incubated with an HRP anti‐HA antibody for 1 h at RT. After another round of washing, the plates were incubated with the TMB peroxidase substrate (BioLegend, USA), and the enzymatic reaction was terminated with 1 M HCl solution. Absorbance was measured at 450 nm to quantify the binding of Nbs to the antigens.

### Surface Plasmon Resonance Analysis

SPR analysis was performed using a Biacore T100 system (GE Healthcare, USA) to determine the affinity of selected Nb to hGRP78. The hGRP78 solution (10 µg mL^−1^) was immobilized onto the flow cell of a CM5 sensor chip (Cytiva, USA) via amine coupling (NHS/EDC) at a flow rate of 10 µL min^−1^. Serially diluted solutions of Nbs were then injected onto the chip surface at a flow rate of 30 µL min^−1^. The association phase was conducted for 120 s, followed by a dissociation phase lasting 300 s. Affinity analysis was performed using multicycle kinetics to determine the binding kinetics and affinity constants.

### Cell Culture

Human colorectal cancer cell lines (HT29, SW620), murine colorectal cancer cell lines (CT26, MC38), murine melanoma cell lines (B16, B16F1, B16F10), murine breast cancer cell line (4T1), murine dendritic cell line (DC2.4), and human embryonic kidney cell (HEK293) were purchased from Cell Bank of the Chinese Academy of Sciences (Shanghai, China) or ATCC (USA). Mouse peritoneal macrophages (PMs) were induced by intraperitoneal injection of 3% thioglycolate and harvested by lavaging the peritoneal cavity with sterile RPMI 1640 (Gibco, USA). HT29, SW620, CT26, MC38, B16, B16F1, B16F10, 4T1, DC2.4, and PMs were cultured in RPMI 1640, while HEK293 cells were cultured in DMEM (Gibco, USA). Complete media were supplemented with 10% fetal bovine serum (Gibco, USA) and 1% penicillin‐streptomycin (Gibco, USA).

### In‐Cell ELISA

To analyze the binding activity of selected Nbs against the csGRP78 protein, cells with high csGRP78 expression were seeded in 96‐well plates and subjected to In‐Cell ELISA analysis. The cells were fixed with 0.25% paraformaldehyde for 10 min, followed by blocking with 3% BSA for 1 h at RT. Serially diluted solutions of HA‐tagged Nbs were added and incubated at RT for 1 h. After three washes with PBS, the plates were incubated with an anti‐HA antibody (Creative Biomart, USA) for 1 h at RT. Subsequently, the plates were washed again and then incubated with the IRDye 680RD goat anti‐mouse IgG secondary antibody (LICORbio, USA) at RT for 1 h. After washing, the plates were imaged using a Sapphire Capture System (Sapphire, USA).

### HSPA5 (GRP78) Knockout using CRISPR‐Cas9 Technology

To generate HSPA5 knockout cells, this work employed the CRISPR‐Cas9 gene editing system. First, single‐guide RNAs (sgRNAs, CCACATACGACGGCGTGATG) targeting the HSPA5 gene were designed and synthesized using a CRISPR design tool. These sgRNAs were then cloned into the lentiCRISPRv2 plasmid vector, which expresses the Cas9 nuclease. The resulting lentiviral constructs were packaged and transfected into the target cells by lentiviral transduction. After 48 h of transduction, cells were selected using puromycin (2.5 µg mL^−1^) for 5–7 days to enrich for successfully edited cells.

### Nanobody Imaging In Vivo

To investigate the in vivo distribution of Nbs, the tumor‐bearing mice were randomly split into two groups and administered 80 µg of Cy5‐labeled Nb C5 or control Nb C9 via intravenous injection. Cy5‐labeled nanobodies were prepared by incubating Nb with Cy5‐Mal at 4 °C overnight at pH 8, using a protein‐to‐maleimide molar ratio of 1:0.5, followed by fluorescence labeling confirmation. At specified time points, mice were imaged using an IVIS instrument (PerkinElmer, Germany). After 24 h post‐injection, tumors and major organs were harvested for ex vivo imaging analysis.

### Immunofluorescence Staining

Cells or tissue slides were fixed using 2% paraformaldehyde or ice‐cold acetone/methanol (4:1), respectively. Nonspecific binding is blocked by 3% BSA or 4% donkey serum before incubation with primary antibodies, including anti‐GRP78 (Proteintech, 11587‐1‐AP), anti‐CALR (Abcam, ab92516), anti‐CD8 (BD Biosciences, 550 281) and anti‐CD68 (BIORAD, MCA1957GA). After washing, samples were incubated with a fluorophore‐conjugated secondary antibody. Following washing steps, samples were exposed to a mounting medium with DAPI for nuclear staining. Fluorescence images were captured using a confocal laser scanning microscopy (Leica SP8, Germany).

### Immunohistochemistry

The tissue microarray from OUTDO BIOTECH, China. Tissue sections were deparaffinized, rehydrated, and subjected to antigen retrieval. Endogenous peroxidase activity was blocked by 0.3% hydrogen peroxide, followed by incubation overnight at 4 °C with primary antibodies, including anti‐GRP78 (Proteintech, 11587‐1‐AP) and Ki67 (Abcam, ab15580). After washing, sections were incubated with appropriate biotin‐labeled secondary antibodies using an ABC kit (Vector, USA) according to the manufacturer's instructions. Finally, sections were dehydrated, cleared, and coverslipped. Images were captured using an inverted light microscope (Leica, Wetzlar, Germany).

### Western Blotting

Cells were lysed using RIPA buffer supplemented with phenylmethylsulfonyl fluoride (PMSF). Following gel electrophoresis, proteins were transferred onto PVDF membranes and blocked with a blocking solution (5% nonfat milk or BSA) for 1 h at room temperature. The membranes were then incubated overnight at 4 °C with primary antibodies diluted according to the manufacturer's instructions. After incubation with the appropriate horseradish peroxidase‐conjugated secondary antibody, Pierce ECL Western Blotting Substrate was used for detection. The following antibodies were used: CHOP (pAb, Proteintech, 15204‐1‐AP), p‐eIF2α^Ser51^ (119a11, Cell Signaling, 3597), Caspase 3 (pAb, Proteintech, 19677‐1‐AP), p‐STING^S365^ (D8F4 W, Cell Signaling, 72 971), STING (ARC57967, Abclonal, A21051), p‐TBK1 (ARC1571, Abclonal, AP1026), TBK1 (ARC0778, Abclonal, A3458), p‐IRF3^S396^ (D6O1 M, Cell Signaling, 29 047, 1:1000), IRF3 (pAb, Abclonal, A11118), and beta‐tubulin (pAb, Proteintech, 10068‐1‐AP).

### Immunotoxin Generation and Characterization

To generate the GRP78‐targeting immunotoxin, the selected Nb C5 was fused to a truncated form of Pseudomonas exotoxin A (PE38) via a (G4S)_3_ linker, resulting in C5‐PE38. A control immunotoxin, C9‐PE38, was also generated. The coding regions of the immunotoxins were cloned into the pET‐15b vector. Recombinant plasmids were then transformed into BL21 (DE3) competent cells and expressed. Following expression, proteins were purified using Ni‐NTA affinity chromatography and the AKTA Pure System. Purified proteins were identified by SDS‐PAGE and western blot analysis.

### Cytotoxicity Assay

The cells were seeded at 3000 cells well^−1^ in 96‐well plates. Upon cell attachment, the medium was replaced with fresh medium containing 2% FBS with various concentrations of C5‐PE38 or C9‐PE38. Cell viability was assessed 48 h later using a Cell Counting Kit‐8 assay.

### Experimental Mouse Models and Treatment Design

All animal experiment procedures were conducted with the guidelines and approval of the Institutional Animal Care and Use Committee (IACUC) of Shenzhen People's Hospital. 6–8‐week‐old female C57BL/6 and 6–8‐week‐old male BALB/c nude (17‐26 g of body weight) were obtained from GemPharmatech Company (China) and maintained housing under specific pathogen‐free (SPF) conditions with 12‐h light/dark cycles.

To create subcutaneous tumor models, HT29 cells (5 × 10^6^ cells/100 µL) were subcutaneously implanted into the right flank of BALB/c nude mice. For the primary melanoma model, B16F10 cells (5 × 10^5^ cells/30 µL) were intradermally injected into the flanks of C57BL/6 mice. To induce metastatic melanoma, B16F10‐Luc cells (2.5 × 10^5^ cells/100 µL) were intravenously injected into C57BL/6 mice via the tail vein. Tumor volume was calculated using the formula: 0.5 × length × width.^2^ Tumor metastasis was monitored using an IVIS imaging system (PerkinElmer, Germany) for bioluminescent signals. The survival endpoint for mice was defined as tumor volume exceeding 1500/2000 mm^3^, or 20% body weight loss.

Preliminary experiments determined that a dose of 0.3 mg kg^−1^ PE38 is both effective and safe for therapeutic application. To assess the therapeutic efficacy of C5‐PE38, tumor‐bearing mice were randomly assigned to groups and intravenously injected with PBS, C5‐PE38 (0.3 mg kg^−1^), or Nb C5 (0.08 mg kg^−1^, equal molar with C5‐PE38) every 2 days for 5 doses. For evaluating the combinational therapeutic potential of C5‐PE38 and anti‐PD1 antibody, C5‐PE38 was administered alone (intravenously, every 2 days) or in combination with 250 µg of anti‐PD1 antibody (Bio X Cell, clone RMP1‐14, intraperitoneally, every 3 days). To elucidate the contribution of the STING pathway in C5‐PE38 therapy, a STING antagonist H151 was used at the initiation of treatment of C5‐PE38 (10 mg kg^−1^, intraperitoneally, daily) to block the STING activity.

### Flow Cytometry Assay

Immunostaining of single‐cell suspensions followed standard protocols. B16F10 tumor tissues were prepared into single suspensions and incubated with anti‐CD16/CD32 (Bioxcell, BE0307) before antibody staining. For T cell and DC analysis, cells were stained with CD45‐PECF594 (BD Biosciences, 562 420), CD3‐FITC (BD Biosciences, 553 062), CD4‐APC (Biolegend, 100 412), CD8a‐APC‐Cy7 (BD Biosciences, 557 654), CD11C‐PE (Biolegend, 117 308), and CD80‐PE‐Cy7 (Biolegend, 104 734). For macrophage analysis, cells were stained with CD45‐PECF594 (BD Biosciences, 562 420), CD11b‐APC‐Cy7 (Biolegend, 101 226), and F4/80‐APC (Biolegend, 123 116). ViaDye Red was used for dead cell exclusion. Samples were analyzed using a full spectrum flow cytometry system (Cytek Biosciences, USA), and data were analyzed using FlowJo (Tree Star, Inc.).

### RNA‐Seq Analysis of Mouse Tumor Tissues

Tumor tissue RNA‐Seq analysis was conducted to explore gene expression profiles. B16F10 tumors were harvested on day 9 from mice treated with either five doses of C5‐PE38 or PBS. Total RNA was extracted and assessed for quality. Libraries were prepared using poly(A) selection and sequenced on an Illumina NovaSeq platform (Novogene). Adapter sequences were initially removed using fastp software prior to subsequent analysis. Reads were aligned to the Mus musculus reference genome sourced from the Ensembl genome browser using STAR software. Gene annotation was performed based on the GTF file from Ensembl. Quantification of gene expression levels was performed in TPM (Transcripts Per Kilobase Million) using RSEM software. Differentially expressed genes were identified using DESeq2, applying criteria of an adjusted p‐value of ≤ 0.05 and a fold change of ≥ 1.2. Gene set enrichment analysis (GSEA) was conducted using the clusterProfiler R package and gene sets derived from the GO Biological Process ontology (GOBP) in MSigDB. Enriched gene sets were identified based on their normalized enrichment scores (NES).

### Quantitative Real‐Time PCR

Total RNA extracted from the same samples used for RNA‐seq was reverse transcribed into cDNA using a reverse transcription kit. qPCR was performed using gene‐specific primers and SYBR qPCR Master Mix (Vazyme, China). The PCR reaction was carried out on a CFX96 RT‐PCR system with the following conditions: 95 °C for 10 min, followed by 40 cycles of 95 °C for 15 s and 60 °C for 1 min. Gene expression levels were normalized to a reference gene (β‐actin), and relative expression was calculated using the 2^−ΔΔCt^ method. Primer sequences are listed in the Table S2, Supporting Information.

### NK Cell Isolation and Identification

Primary NK cells were isolated from mouse spleens using a negative selection method (480049, BioLegend). Briefly, spleen tissue was mechanically dissociated, and a biotinylated antibody cocktail targeting non‐NK cell markers was added to the cell suspension. Streptavidin‐coated magnetic beads were then used to bind and remove non‐NK cells. The magnetically labeled non‐NK cells were retained in a magnetic column, while the untouched NK cells flowed through and were collected. The isolated NK cells were subsequently stained with specific antibodies, including CD49b and CD3, for flow cytometry analysis to assess NK cell purity. Flow cytometric analysis was performed to identify and quantify the NK cell population.

### STING Pathway Detection

B16F10 cells were seeded and treated with C5‐PE38 at concentrations of 100 or 500 nM. After 24 h, the culture medium was harvested and centrifuged. The resulting supernatants were then incubated with pre‐seeded mouse peritoneal macrophages or DC2.4 cells. After 4 h of stimulation, cell lysates were collected for the determination of STING pathway indicators with western blot analysis (STING, p‐STING, p‐TBK1 and p‐IRF3). To degrade DNA, 2 U mL^−1^ of DNase I was added to the supernatant and incubated at 37 °C for 2 h. EDTA (1 mM) was added to the supernatant and heated at 95 °C for 10 min to inactivate the DNase I. To suppress the STING pathway, DC2.4 cells were pre‐treated with H151 (5 µM), and the drug concentration was maintained throughout the subsequent supernatant stimulation. After 4 h of stimulation, cell lysates were collected for western blot analysis.

### Statistical Analysis

Data are presented as mean ± standard error of measurement (SEM) unless stated otherwise. The number of biologically independent replicates (n) is indicated for all datasets. Statistical analyses were conducted using GraphPad Prism 8.0. Statistical significance was determined using unpaired Student's *t*‐test for comparisons between two groups or analysis of variance (ANOVA) for comparisons among multiple groups, as specified in the figure legends. Survival curves were evaluated using the log‐rank Mantel‐Cox test. A *p*‐value of less than 0.05 was considered statistically significant, with asterisks denoting significant differences (*p < 0.05, **p < 0.01, ***p < 0.001, and ****p < 0.0001).

### Ethical Statement

All animal experiment procedures were conducted with the guidelines and approval of the Institutional Animal Care and Use Committee (IACUC) of Shenzhen People's Hospital (approval number: AUP‐230224‐LZJ‐543‐01).

## Conflict of Interest

The authors declare no conflict of interest.

## Author Contributions

H.W., R.Z., C.X., and L.D., contributed equally to this work. Huifang Wang: conceptualization, data curation, investigation, writing – original draft. Runhua Zhou: data curation, investigation. Chengchao Xu: data curation, investigation. Lingyun Dai: data curation, investigation. Liuhai Zheng: data curation, investigation. Chunjin Fu: data curation. Guangwei Shi: data curation. Xiaolong Xu: data curation. Rui Hou: data curation. Jingwei Wang: data curation. Yang Li: data curation. Jinpeng Cen: data curation. Le Yu: data curation. Yawei Liu: data curation. Yilei Li: funding acquisition. Qingfeng Du: writing – review & editing, funding acquisition. Zhijie Li: conceptualization, writing – review & editing, funding acquisition. Jigang Wang: funding acquisition.

## Supporting information



Supporting Information

## Data Availability

The data that support the findings of this study are available from the corresponding author upon reasonable request.
