# Supplementary material for: GRP78 Nanobody‐Directed Immunotoxin Activates Innate Immunity Through STING Pathway to Synergize Tumor Immunotherapy
Source: Adv Sci (Weinh). 2025 Mar 26;12(19):2408086. doi: 10.1002/advs.202408086 (PMC12097070; doi:10.1002/advs.202408086)
Supplement: Supplementary file 1 — Supporting Information [file ADVS-12-2408086-s001.docx]

**GRP78 Nanobody-directed Immunotoxin Activates Innate Immunity through STING Pathway to Synergize Tumor Immunotherapy**

*Huifang Wang, Runhua Zhou, Chengchao Xu, Lingyun Dai, Rui Hou, Liuhai Zheng, Chunjin Fu, Guangwei Shi, Jingwei Wang, Yang Li, Jinpeng Cen, Xiaolong Xu, Le Yu*, Yilei Li*, Jigang Wang*, Qingfeng Du*, Zhijie Li**

H. Wang, C. Xu, L. Dai, R. Hou, L. Zheng, C. Fu, Y. Li, X. Xu, , J. Wang, Z. Li

Department of Critical Care Medicine, Guangdong Provincial Clinical Research Center for Geriatrics, Shenzhen Clinical Research Centre for Geriatrics, Department of Nuclear Medicine Shenzhen People’s Hospital (The First Affiliated Hospital, Southern University of Science and Technology; The Second Clinical Medical College, Jinan University)

Shenzhen, Guangdong 518020, China.

E.mail: Zhijie Li, [li.zhijie@szhospital.com](mailto:li.zhijie@szhospital.com); Jigang Wang, [wangjigang@u.nus.edu](mailto:wangjigang@u.nus.edu)

H. Wang

Post-doctoral Scientific Research Station of Basic Medicine

Jinan University

Guangzhou 510632, China

R. Zhou, J. Wang, L. Yu, Y. Li

Clinical Pharmacy Center

Nanfang Hospital, Southern Medical University

Guangzhou 510515, Guangdong, China.

E.mail: Yilei Li, [leilei1975@hotmail.com](mailto:leilei1975@hotmail.com); Le Yu, [yule0423@gmail.com](mailto:yule0423@gmail.com)

R. Hou

Harry Perkins Institute of Medical Research, QEII Medical Centre and Centre for Medical Research

The University of Western Australia

Nedlands, WA 6009, Australia

G. Shi

Department of Neurosurgery & Medical Research Center

Shunde Hospital, Southern Medical University (The First People’s Hospital of Shunde Foshan)

Guangzhou 510515, China.

J. Cen

Department of Urology

Nanfang Hospital, Southern Medical University

Guangzhou, Guangdong 510515, China

L. Yu, J. Wang, Q. Du

School of Traditional Chinese Medicine and School of Pharmaceutical Sciences, Guangdong Provincial Key Laboratory of New Drug Screening, School of Pharmaceutical Sciences

Southern Medical University

Guangzhou 510515, Guangdong, China.

E.mail: Qingfeng Du, [dqf1689@smu.edu.cn](mailto:dqf1689@smu.edu.cn)

J. Wang

State Key Laboratory for Quality Ensurance and Sustainable Use of Dao-di Herbs, Artemisinin Research Center, Institute of Chinese Materia Medica

China Academy of Chinese Medical Sciences

Beijing 100700, China.

J. Wang

State Key Laboratory of Antiviral Drugs, School of Pharmacy

Henan University

Kaifeng 475004, China.

**Supplementary figures**


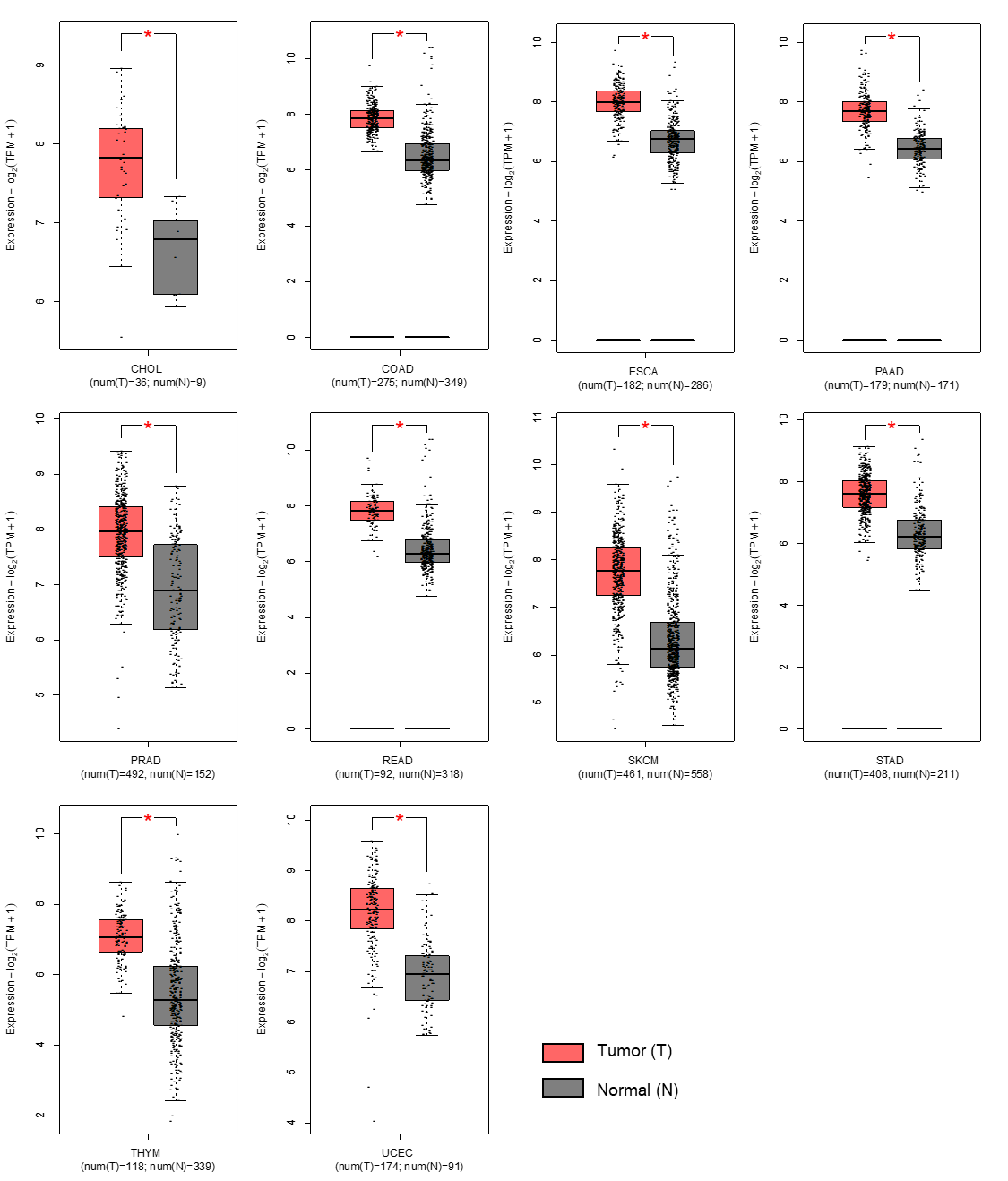


**Figure S1. Expression analysis of HSPA5 in TCGA dataset**. The normalized expression levels of HSPA5 were assessed across a broad range of cancer types using data from the TCGA and GTEx databases. Statistical significance (T-test) is indicated as **P* < 0.05. CHOL, Cholangiocarcinoma; COAD, Colon Adenocarcinoma; ESCA, Esophageal Carcinoma; PAAD, Pancreatic Adenocarcinoma; PRAD, Prostate Adenocarcinoma; READ, Rectum Adenocarcinoma; SKCM, Skin Cutaneous Melanoma; STAD, Stomach Adenocarcinoma; THYM, Thymoma; UCEC, Uterine Corpus Endometrial Carcinoma.


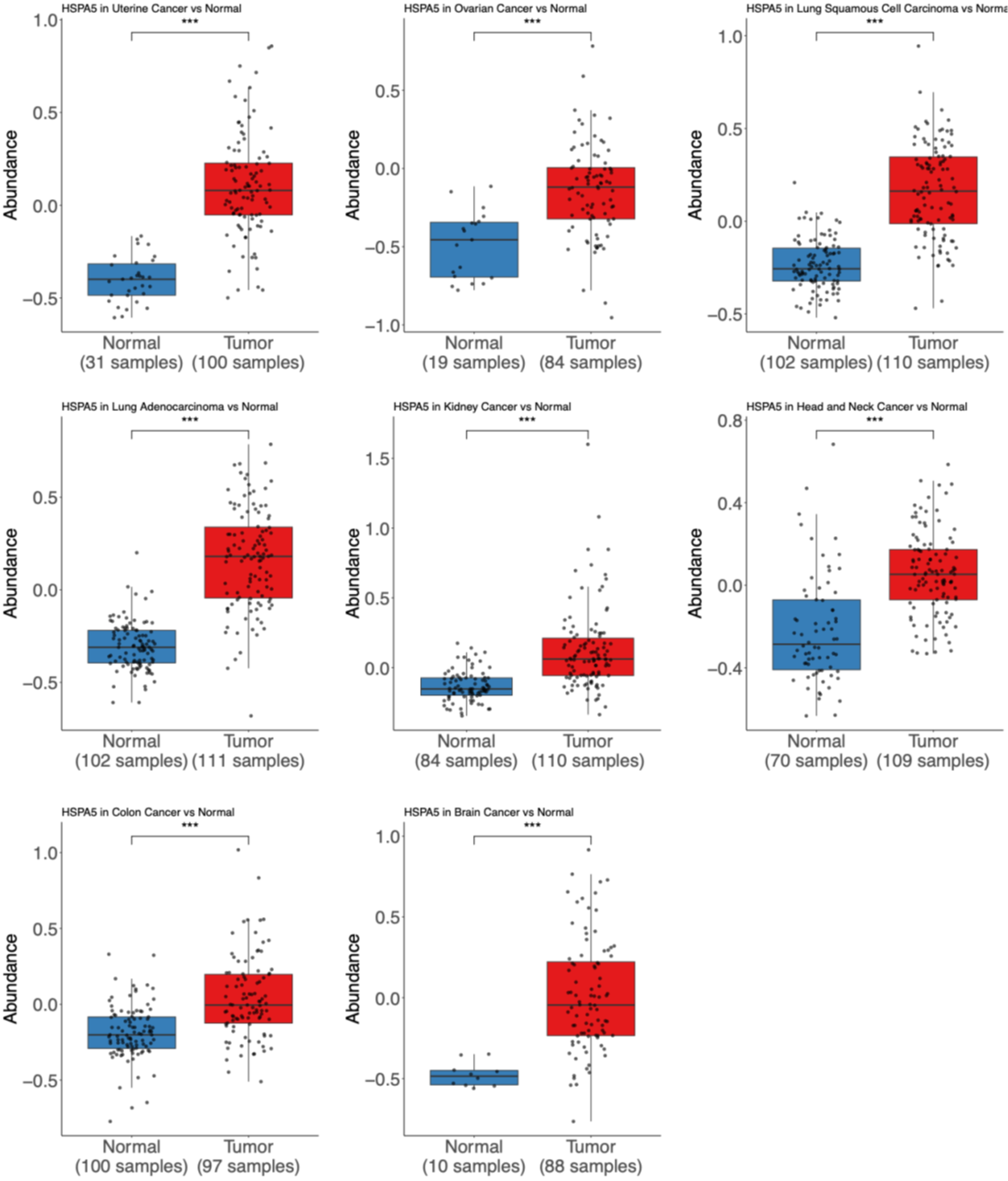


**Figure S2. Comparison of HSPA5 relative protein abundances between tumor and normal tissues**. Using the CPTAC dataset, the relative protein abundances of HSPA5 in primary tumor tissues compared to their corresponding normal tissues. The analysis underscores the differential expression of HSPA5 protein across various cancer types. Statistical significance (T-test) is indicated as follows: **P* < 0.05; ***P* < 0.01; ****P* < 0.001.


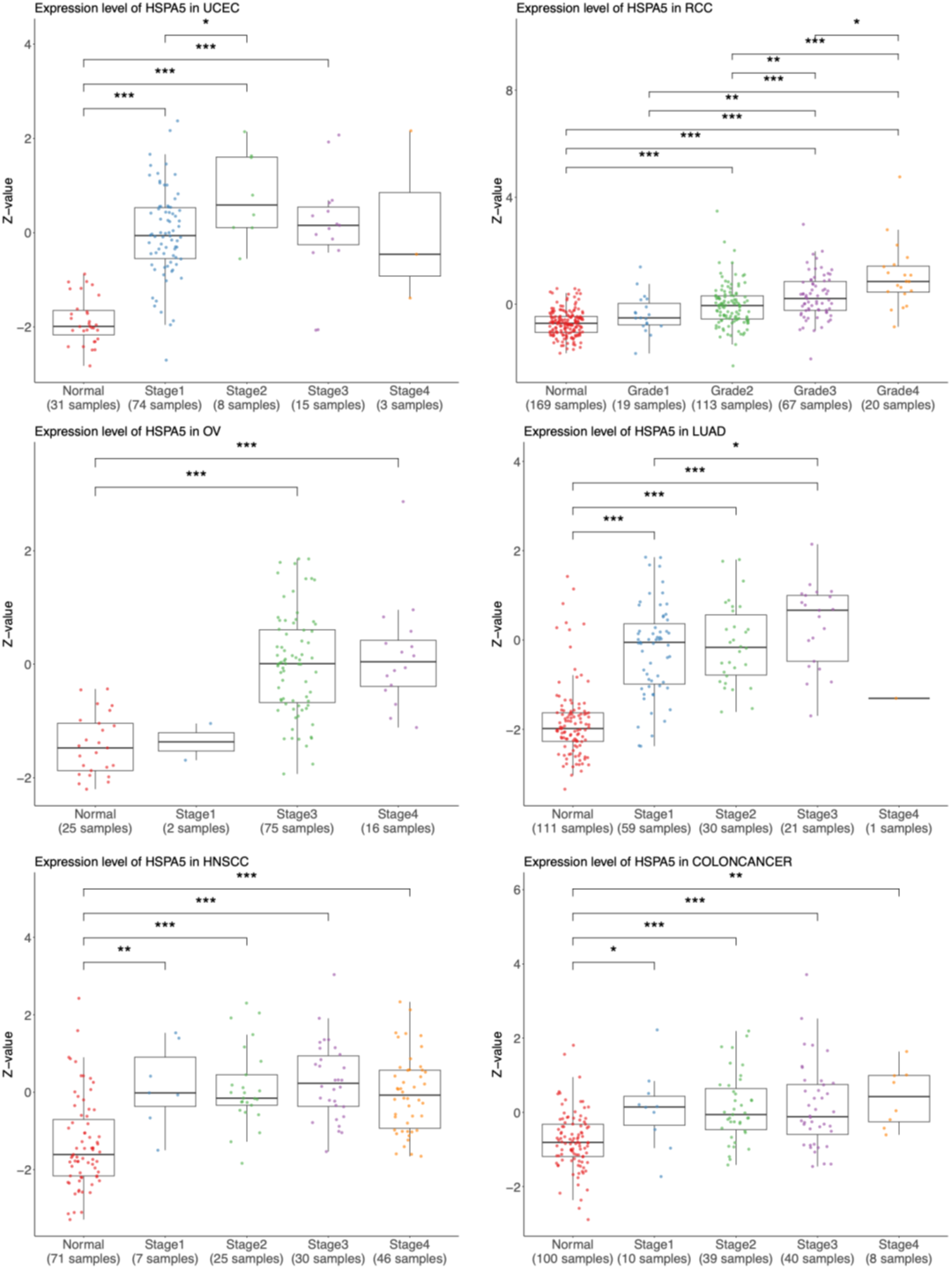


**Figure S3. Pathological analysis of HSPA5 protein in various cancers across different pathological stages**. HSPA5 protein expression across different pathological stages in six cancer types, utilizing data from the CPTAC dataset. Z-values represent the standardized differences between the means of the tissue groups. Statistical significance (T-test) is indicated as follows: **P* < 0.05; ***P* < 0.01; ****P* < 0.001. UCEC, Uterine Corpus Endometrial Carcinoma; RCC, Renal Cell Carcinoma; OV, Ovarian Serous Cystadenocarcinoma; LUAD, Lung Adenocarcinoma; HNSCC, Head and Neck Squamous Cell Carcinoma.


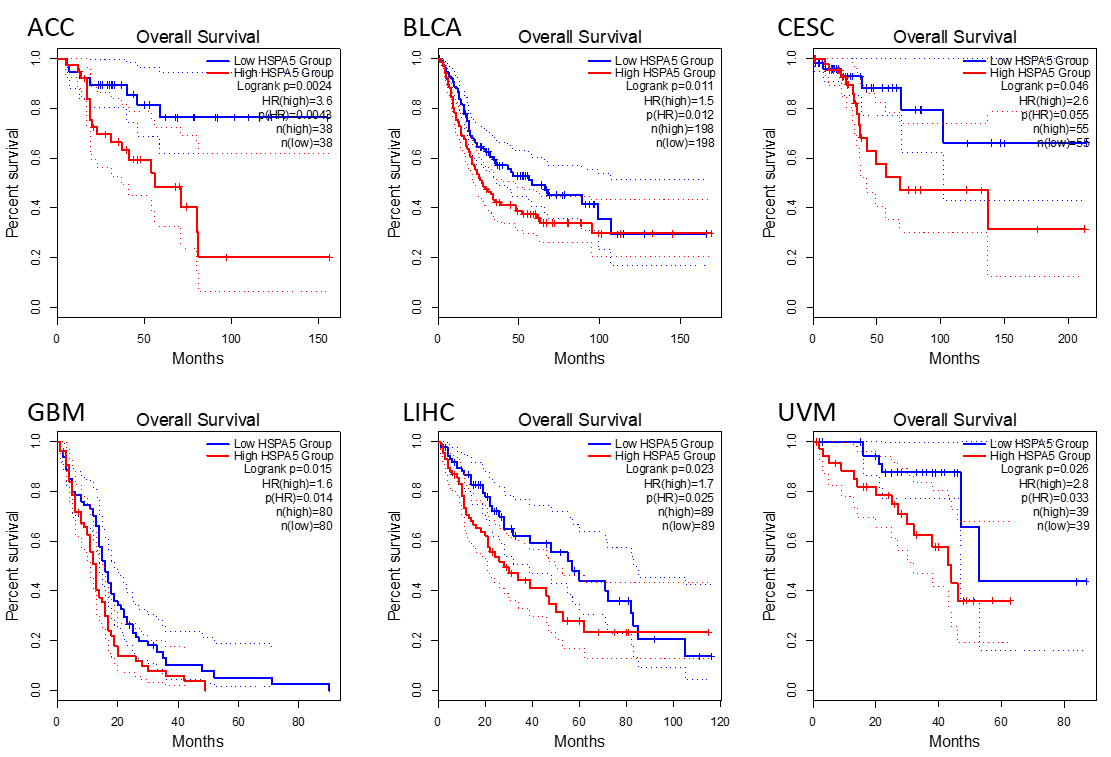


**Figure S4. HSPA5 expression and prognostic impact on survival.** The relationship between HSPA5 expression levels and patient survival outcomes was examined, utilizing Gepia2 and TCGA data. Patients were categorized into high and low-expression groups based on the median expression level of HSPA5. Kaplan-Meier survival curves were generated to evaluate the impact of HSPA5 expression on overall survival (OS) across various cancer types. Statistical significance was assessed using the log-rank test. ACC, Adrenocortical Carcinoma; BLCA, Bladder Urothelial Carcinoma; CESC, Cervical Squamous Cell Carcinoma and Endocervical Adenocarcinoma; GBM, Glioblastoma Multiforme; LIHC, Liver Hepatocellular Carcinoma; UVM, Uveal Melanoma.


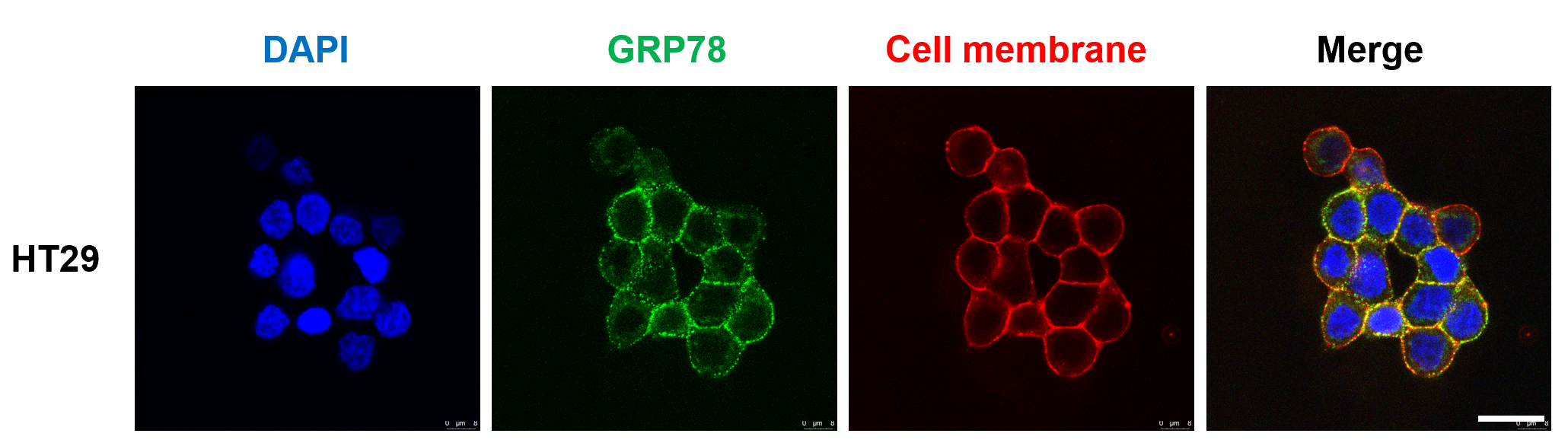


**Figure S5. Representative immunofluorescence images of csGRP78 staining in HT29 cells.** HT29 cells were stained with an anti-GRP78 antibody (green) and a cell membrane dye, Cellbrite (red). The fluorescence images showed the localization of csGRP78 on the cell surface. Scale bar, 20 μm.

**Figure S6. Screening of GRP78-binding nanobodies (Nbs) using a naïve alpaca Nb phage library.** Deep sequencing results of the third round of phage eluates from each antigen screening. The vertical axis represents sequence counts, and the percentage of sequence frequencies is indicated on the bar graph. Nbs with sequence frequencies ≥ 0.9% were selected for further identification, highlighted in blue.

**Figure S7. ELISA analysis assessing the binding affinity of 10 Nbs and an irrelevant control Nb (C9) to BSA.**

**Figure S8.** **Evaluation of nanobody binding specificity to GRP78 in GRP78 knockout cells.** (**a**) GRP78 knockout in B16 cells was achieved using lentivirus-based CRISPR-Cas9 gene editing technology and the efficiency was determined by western blot. (**b**) Comparison of the binding specificity of Nbs NBD1 and C5 to GRP78 in GRP78 knockout and control cells using dot blot analysis. Protein extracts from both cell lines were spotted onto a nitrocellulose (NC) membrane and incubated with the nanobodies (1 μg/mL) (mean ± SEM, n = 3, unpaired two-tailed Student’s t-test). (**c**) In-cell ELISA analysis detecting the binding activity of Nbs NBD1 and C5 to GRP78 in GRP78 knockout and control B16 cells. Cells were fixed, followed by incubation with the nanobodies (mean ± SEM, n = 3, two-way ANOVA with Tukey’s multiple comparisons). **P* < 0.05, ***P* < 0.01, ****P* < 0.001, *****P* < 0.0001.

**Figure S9. SDS-PAGE analysis followed by fluorescence imaging confirmed the successful conjugation of each Nb with Cy5-Mal**. The conjugation was achieved by incubating Nbs with Cy5-Mal at a 1:0.5 protein to maleimide molar ratio at 4 °C overnight at pH 8.

**Figure S10. C5-PE38 demonstrates low cytotoxicity in MC38 tumor cells with low csGRP78 expression**. Cells were treated with C5-PE38 or C9-PE38 for 48 h. Cell viability was assessed using the CCK8 assay (mean ± SEM, n = 5).


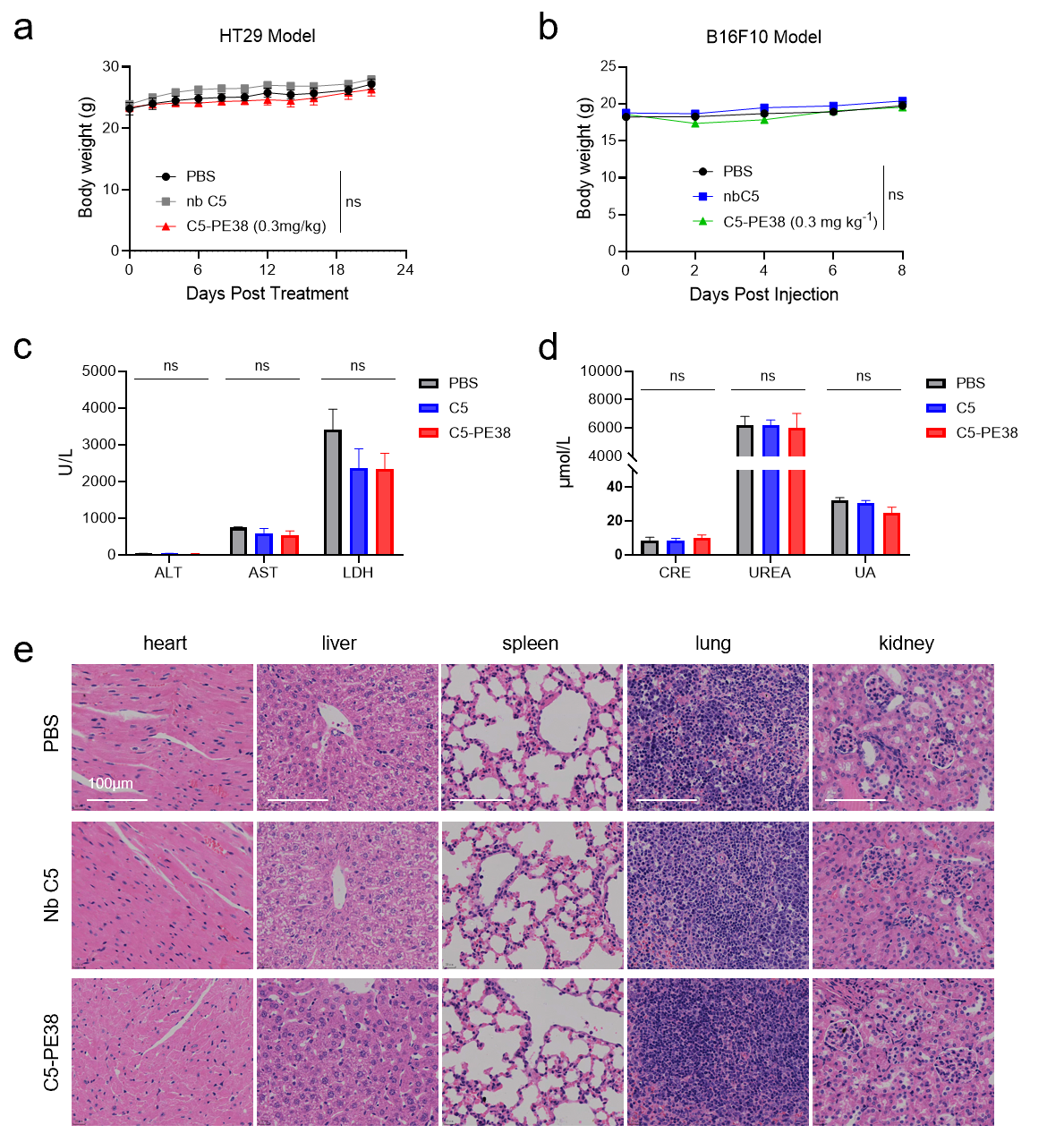


**Figure S11. In vivo safety assessment. (a**-**b)** Changes in body weight of mice following various treatments in HT29 model (mean ± SEM, n = 6) and B16F10 model (mean ± SEM, n = 5). Tumor-bearing mice received five doses of PBS, Nb C5, or C5-PE38, respectively. **(c)** Evaluation of liver function in B16F10 tumor-bearing mice, including alanine aminotransferase (ALT), aspartate aminotransferase (AST), and lactate dehydrogenase (LDH) (mean ± SEM, n = 4). (**d**) Assessment of kidney function in B16F10 tumor-bearing mice, including creatinine (CRE), urea and uric acid (UA) (mean ± SEM, n = 4). B16F10-bearing mice received five doses of PBS, Nb C5, or C5-PE38 and sacrificed on day 9. (**e**) Histopathological examination of heart, liver, spleen, lung, and kidney tissues from B16F10-bearing mice treated with C5-PE38 or Nb C5 showed no differences compared to control mice (n = 3). Scale bars, 100 μm**.** Statistical significance was determined by two-way ANOVA with Tukey’s multiple-comparisons test (**a**, **b**) and one-way ANOVA with Tukey’s multiple-comparisons test (**c**, **d**). n.s., no significance.

**Figure S12. Flow cytometry gating strategies for TME analysis t in the B16F10 tumor model.**


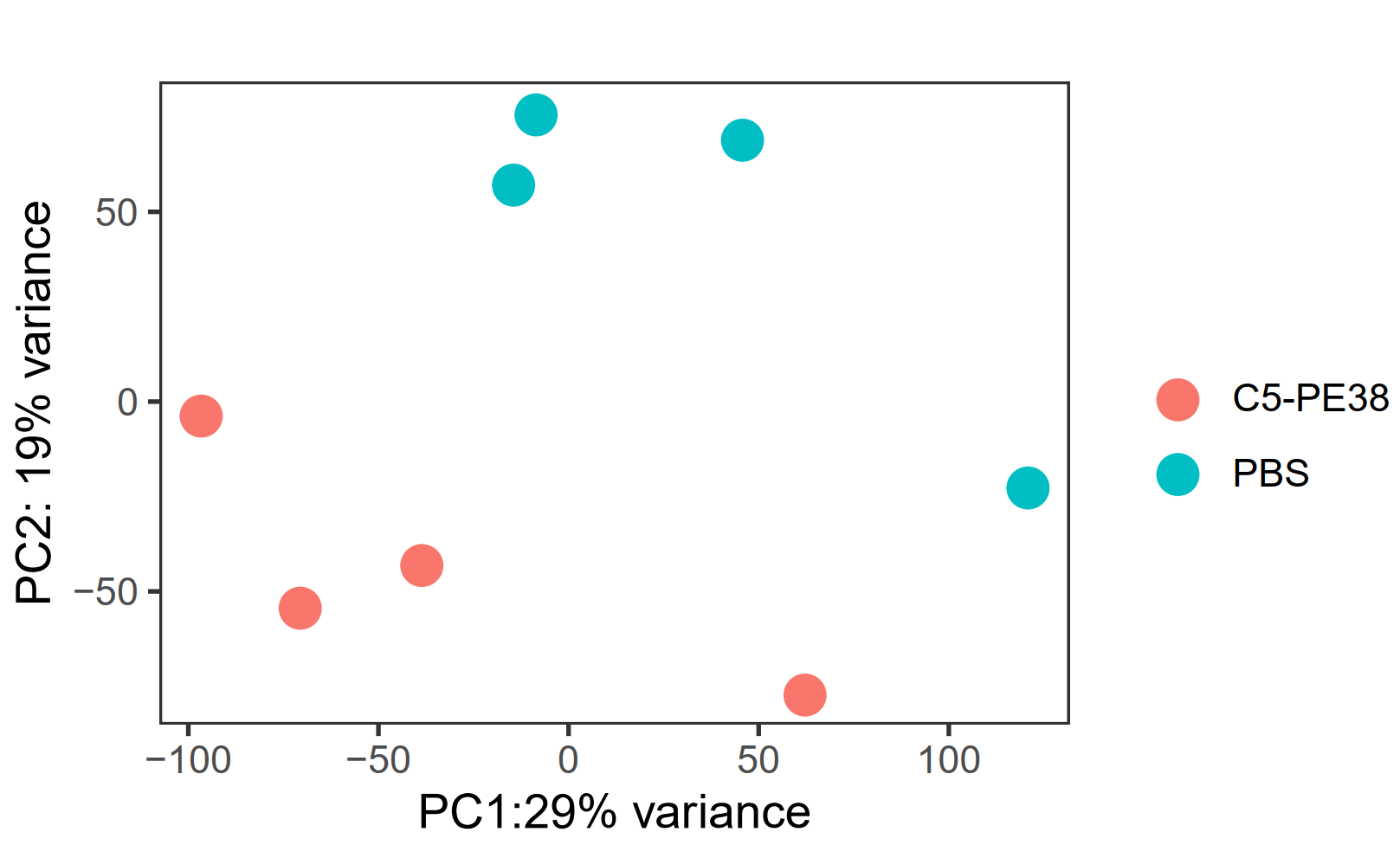


**Figure S13**. Principal component analysis (PCA) analysis of the RNA-Seq data.


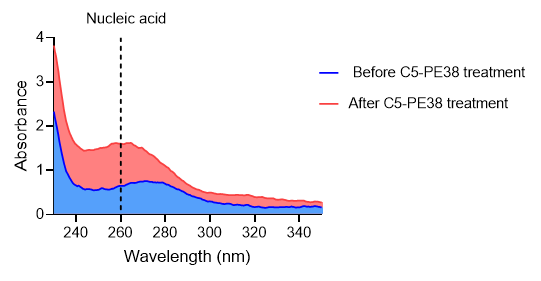


**Figure S14**. **The absorbance of supernatants from B16F10 cells treated with C5-PE38 was measured by spectrophotometry**. The absorption peak corresponding to nucleic acids (260 nm) was indicated by dashed lines.

**Figure S15. Western blot and quantitative analysis of STING pathway activation in peritoneal macrophages and DC2.4 cells**. (**a**) Quantitative analysis of the relative expression levels of p-STING/STING, p-TBK1/TBK1, and p-IRF3/IRF3 in peritoneal macrophages and DC2.4 cells as shown in Figure 6g (mean ± SEM, n = 3). (**b**) Quantitative analysis of the relative levels of p-STING/STING, p-TBK1/TBK1, and p-IRF3/IRF3 in DC2.4 cells as shown in Figure 6h (mean ± SEM, n = 3). Statistical significance was assessed by one-way ANOVA with Tukey’s multiple comparisons. **P* < 0.05, ***P* < 0.01, ****P* < 0.001.


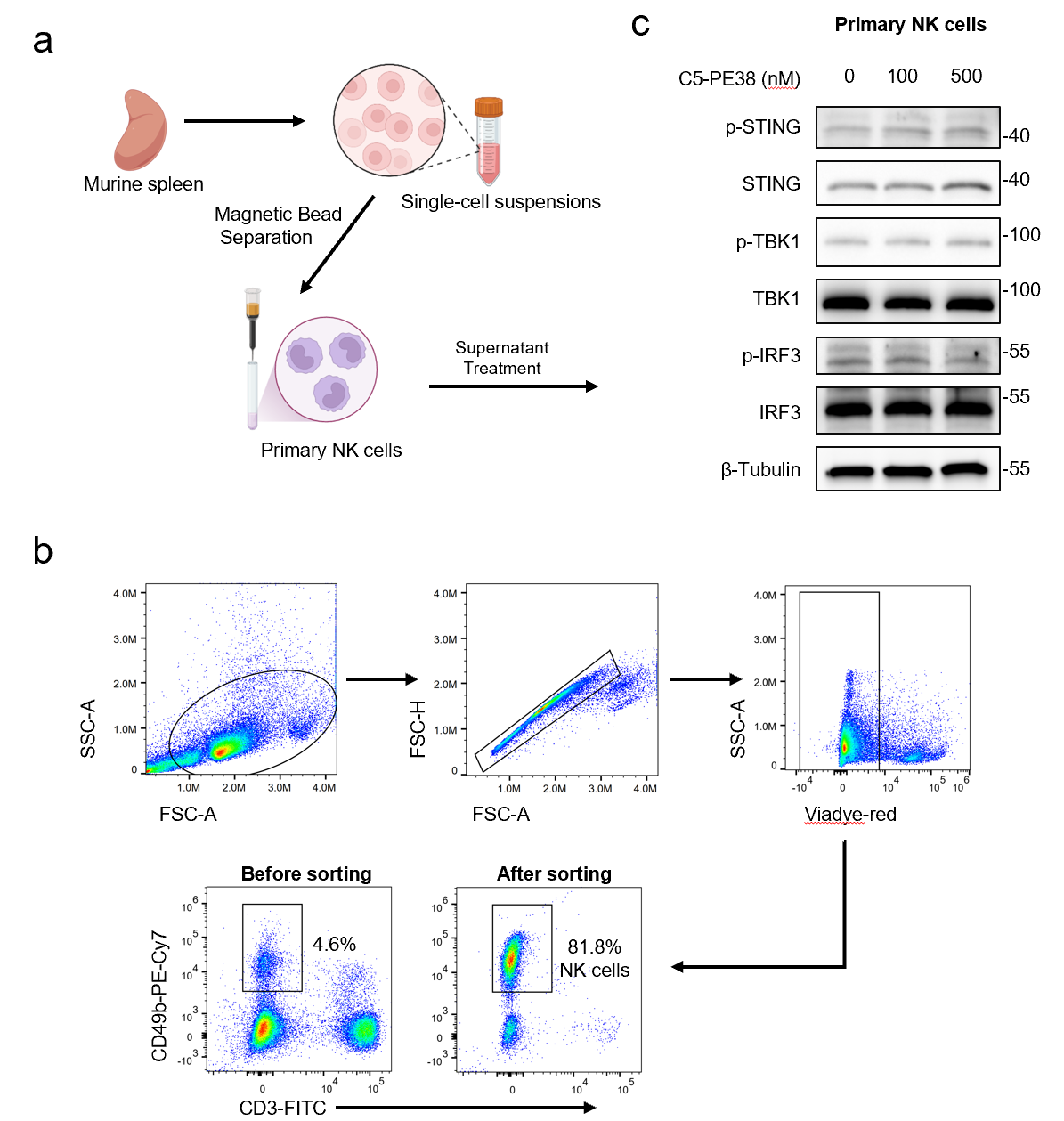


**Figure S16. Isolation and STING pathway detection in primary mouse NK cells.** (**a**) Schematic diagram illustrating the isolation of primary mouse NK cells from the spleen. (**b**) Flow cytometry analysis of NK cell populations before and after sorting, showing changes in NK cell purity following sorting based on specific markers (CD49b and CD3). (**c**) Detection of STING pathway activation in primary NK cells following stimulation with C5-PE38 conditioned medium. NK cells were cultured with supernatant from C5-PE38-treated cells for 4 hours, and activation of the STING signaling pathway was assessed by western blotting.

**Figure S17.** **C5-PE38 treatment indirectly enhances IFNβ** **and TNF-α production in dendritic cells and macrophages**. ELISA analysis of IFN-β (**a**) and TNF-α (**b**) levels in cell culture supernatants from tumor cells, DC2.4, and peritoneal macrophages. Cell supernatants were collected from tumor cells 24 hours after C5-PE38 treatment, and from DC2.4 and peritoneal macrophages 24 hours after stimulation with C5-PE38-treated tumor cell supernatants. The concentrations of IFN-β and TNF-α in these supernatants were measured using ELISA (mean ± SEM, n = 3). Statistical significance was assessed by unpaired two-tailed Student’s t-test. **P* < 0.05, ***P* < 0.01, ****P* < 0.001.


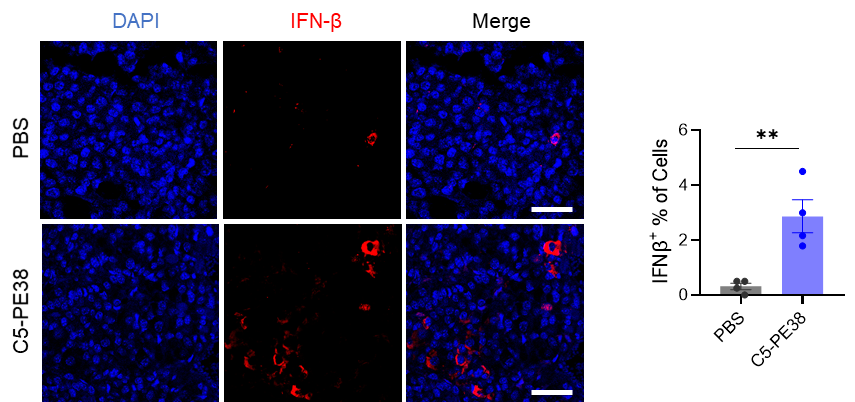


**Figure S18.** **C5-PE38 treatment enhances IFN-β production within the tumor microenvironment**. Representative immunofluorescence images and quantification of IFN-β staining in tumor tissues from the B16F10 tumor model (mean ± SEM, n = 4). Scale bars, 50 μm. Statistical significance was assessed by unpaired two-tailed Student’s t-test. **P* < 0.05, ***P* < 0.01, ****P* < 0.001.


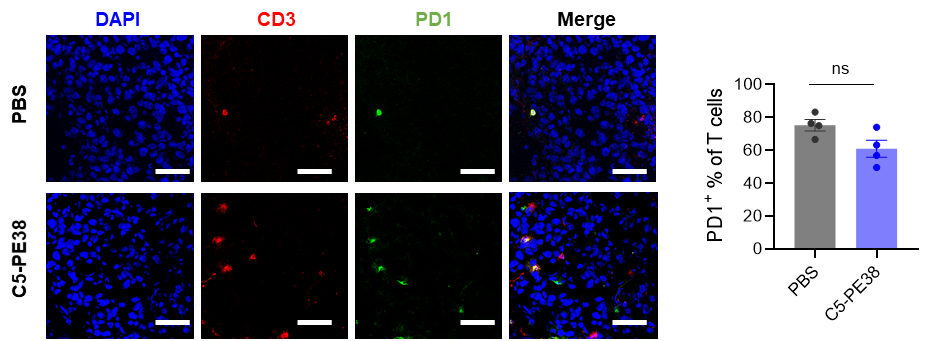


**Figure S19.** **PD-1 is expressed on intratumoral T cells in B16F10 tumors.** Representative immunofluorescence images and quantitative analysis of CD3^+^ and PD-1^+^ expression in B16F10 tumor tissue (mean ± SEM, n = 4). CD3^+^ (red) and PD-1^+^ (green) co-expression were indicated yellow in the merged color overlay. Cells were counter-stained with DAPI. Scale bars, 50 μm. Statistical significance was determined using an unpaired two-tailed tudent’s t-test. n.s., no significance.

**Figure S20. Anti-GRP78 PE38 immunotoxin and α-PD1 combination elicits robust systematic immunity against primary and metastatic melanomas.** (**a**) Individual tumor growth curves of B16F10 tumors from Figure 7b (n = 6-7 per group). (**b**) Individual tumor growth curves of B16F10 tumors from Figure 7e (n = 6 per group).

**Figure S21.** **Changes in body weight of mice following various treatments in two melanoma models**. (**a**) Mice were treated as indicated in Figure 7a and body weight was recorded every two days (mean ± SEM, n = 6-7 per group). (**b**) Mice were treated as indicated in Figure 7g and body weight was recorded every two days (mean ± SEM, n = 7 per group). (**c**) Body weight of individual mice with B16F10-Luc lung metastases throughout the experiment related to Figure 7g (n = 7). Statistical significance was determined by two-way ANOVA with Tukey’s multiple-comparisons test (**a**, **b**). n.s., no significance.

**Supplementary tables**

**Table S1. Nanobody CDR Sequence**

| **Nanobody** | **CDR1** | **CDR2** | **CDR3** |
| --- | --- | --- | --- |
| Nb C5 | DYYAIG | GVSCISSAGVLTN | AAADARQLKVRQCLSSNAYT |
| Nb SBD1 | RGTHMA | WVALLSRYGSTH | ARGGPFVS |
| Nb SBD2 | RGTHMA | WVALLSRYGSTH | RGFG |
| Nb SBD3 | SGYFMG | FVAGISRSGVNTY | NARPWRWSPNSQ |
| Nb SBD4 | INYRMG | FVAAISWNGGSTY | YAQRGWEE |
| Nb C1 | SGNTMA | LVASITTSGITN | NAQATSVLLPRVTRN |
| Nb NBD1 | NPALVG | MVAMISISGNTN | KKLPFG |
| Nb NGS2 | SRYTMA | FVARITWSGSRY | AAQGVSATATFLRGYA |
| Nb NGS3 | RGYIMG | FVAAISWSGSITE | RARRGIVPLYD |
| Nb NGS4 | SWYVMG | LVATISSGGSRN | AARRVRSGLLLFNPQGYN |
| Nb NGS5 | SRYLMG | FVASITAISWSGASTR | AARGSWGTYYVAPDRYA |

**Table S2. Sequences of PCR primers**

| **Gene** | **Forward primer (5' to 3')** | **Reverse primer (5' to 3')** |
| --- | --- | --- |
| m_β-Actin | GGCTGTATTCCCCTCCATCG | CCAGTTGGTAACAATGCCATGT |
| m_Cxcl10 | CCAAGTGCTGCCGTCATTTTC | GGCTCGCAGGGATGATTTCAA |
| m_Ccl5 | GCTGCTTTGCCTACCTCTCC | TCGAGTGACAAACACGACTGC |
| m_Ifnb1 | CAGCTCCAAGAAAGGACGAAC | GGCAGTGTAACTCTTCTGCAT |
| m_Ifit2 | AGTACAACGAGTAAGGAGTCACT | AGGCCAGTATGTTGCACATGG |
| m_Tnf | GGTCCCCAAAGGGATGAGAAGT | TTGCTACGACGTGGGCTACA |
